# Ultrathin Soft Wearable Sensor Materials and Structures: A Review of Current Trends and Prospectives

**DOI:** 10.1021/acsami.5c14329

**Published:** 2025-10-21

**Authors:** Jinyoung Kim, Dong-hee Kang, Min Sub Kwak, Geonyoung Jung, Jeeyoon Kim, Sehyun Park, Hyunhyub Ko, Vladimir V. Tsukruk

**Affiliations:** † School of Materials Science and Engineering, 1372 Georgia Institute of Technology, Atlanta, Georgia 30332, United States; ‡ School of Energy and Chemical Engineering, 131639 Ulsan National Institute of Science and Technology, Ulsan Metropolitan City 44919, South Korea

**Keywords:** ultrathin composite film, skin-electronics interface, wearable sensors, electrical/optical sensing, human and healthcare monitoring

## Abstract

Ultrathin wearable sensors have emerged as a transformative platform for next-generation health and performance monitoring, offering intimate integration with human skin for real-time physiological and biochemical sensing based upon electrophysical and electrochemical response at human–sensor interfaces. These sensory systems, often composed of micrometer-to-nanometer-thick functional responsive materials, achieve seamless integration at skin-electronics interfaces by mimicking the dynamic and mechanical properties of human skin. Recent advances in material synthesis, nanoscale engineering, and structural design have enabled novel sensors that are not only stretchable and breathable but also robust, biocompatible, and highly responsive. This review highlights the fundamental materials principles governing skin-conformal interactions, explores various material systems, including planar, porous, and hybrid architectures, and outlines state-of-the-art developments in smart adhesives, wearable ultrathin sensors, responsive behavior, true conformability, and printed sensors. We discuss the broad spectrum of current and prospective applications, from tactile and electrophysiological sensing to biochemical and multimodal wearable devices, as well as key challenges, existing trends, and future prospects.

## Graphical abstract

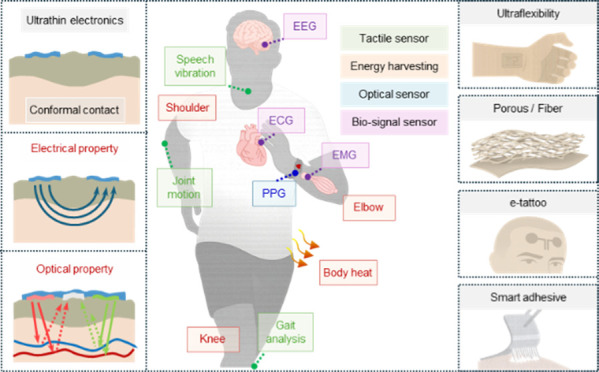

## 1 Introduction

Wearable sensors that adhere directly to human skin have rapidly advanced in the past decade.^1–3^ They promise continuous, high-fidelity monitoring of tactile stimuli and physiological signals (e.g., heart rate, brain activity, muscle activity, eye movement, temperature, etc.) in an unobtrusive manner due to novel materials design achievements.^4^ Unlike conventional rigid metal electronics, ultrathin, ultrasoft sensory materials can intimately laminate onto the skin, virtually melding with its complex surface texture and matching dynamic properties. By making these sensors extremely thin down to single-digit micrometers, submicrons, or even at the nanoscale (hundreds of nm) and using soft materials, researchers minimize the mechanical and sensory mismatch at human–synthetic interfaces. Ultrathin wearable sensors offer unique advantages in conformability, mechanical compatibility, and user comfort—features essential for long-term, unobtrusive, and dynamic operation.

As illustrated in Figure 1a, ultrathin wearable sensors might exhibit clear advantages over their thicker counterparts across multiple domains of skin-interfaced sensing.^5^ In terms of mechanical interaction, conventional thick electronics often form incomplete, loose contact with the skin, resulting in gaps, motion artifacts, and reduced signal fidelity.^6^ In contrast, ultrathin electronic materials conform intimately to the microtopography of the skin, such as ridges, grooves, and soft curvatures, ensuring stable, continuous signal acquisition without additional adhesives or mechanical constraints due to stiff contacts.

**Figure 1:**
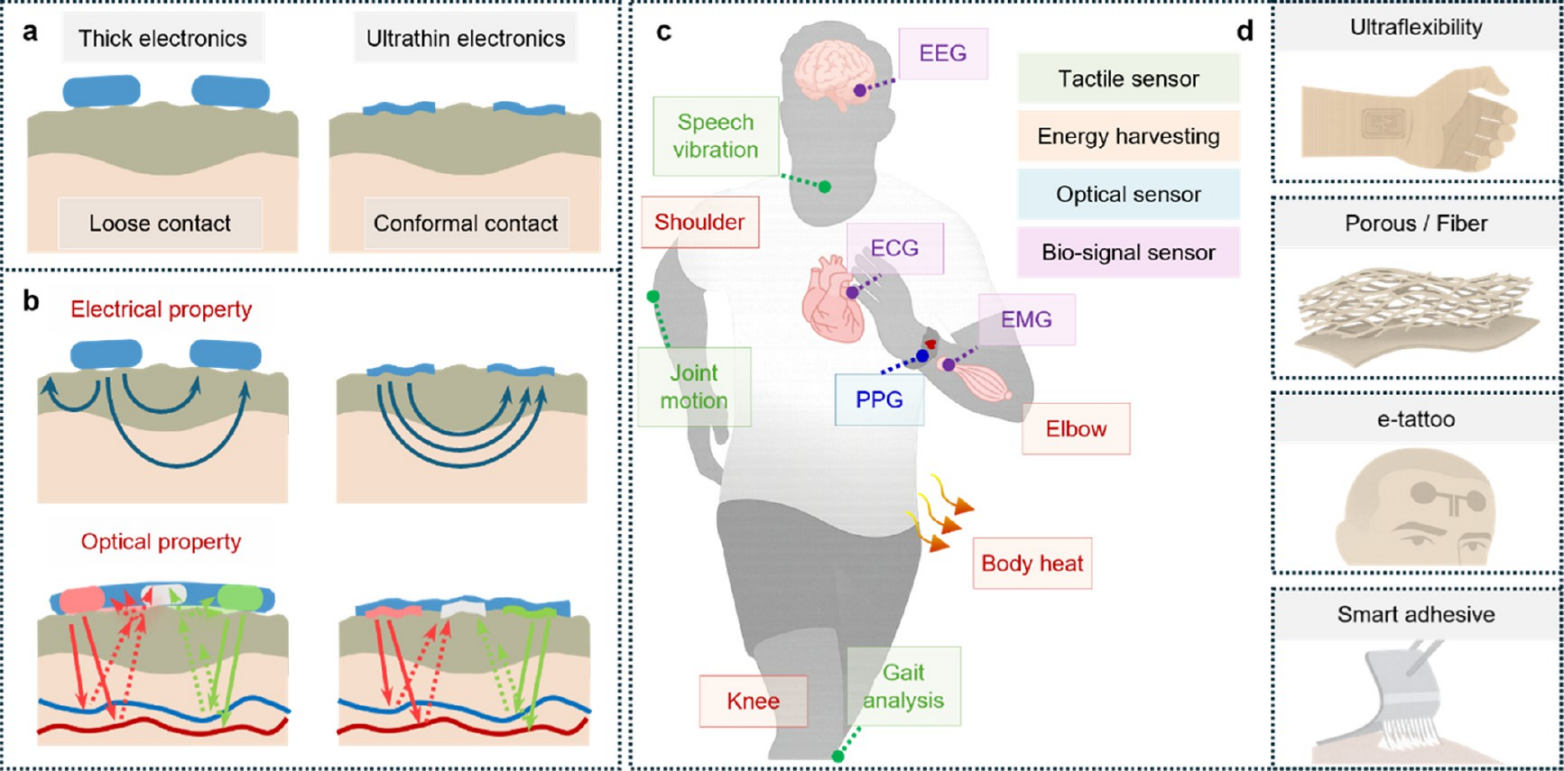
Overview of ultrathin soft wearable sensor applications. (a) Comparison of thick and ultrathin wearable sensors, in terms of skin contact, (b) signal transduction, and device functionalities. Thick sensors exhibit loose contact with skin, resulting in degraded electrical and optical signal transmission, while ultrathin devices conform seamlessly to skin microtextures, (c) enabling improved tactile sensing (joint motion, gait analysis, speech vibration), different biosignal acquisition (EEG, ECG, EMG), optical sensing (PPG), and energy harvesting (shoulder, elbow, knee). (d) Various structural platforms, including ultraflexible substrates, porous/fiber networks, e-tattoos, and smart adhesives, support multifunctionality and biocompatible integration across different body regions.

The conformal interfaces enhance both electrical response and optical signal transmission (Figure 1b). For electrical sensing, improved skin-sensor contact minimizes interfacial impedance, which is critical for high-resolution biopotential recordings such as electroencephalogram (EEG), electrocardiogram (ECG), electromyogram (EMG), and electrooculogram (EOG).^7^ Similarly, in optical sensing like photoplethysmography (PPG), conformality reduces optical scattering and reflection artifacts, enabling more accurate detection of pulse and blood oxygen levels. Ultrathin sensors also significantly reduce noise arising from micromovements and triboelectrical interference, thereby maintaining higher signal stability during intense motion or sweat-induced environmental changes.

As shown in Figure 1c, ultrathin wearable sensors can be applied across diverse body regions to monitor a variety of physiological signals. EEG sensors on the scalp capture brain activity, ECG on the chest measures cardiac signals, and EMG on the limbs detects muscle activity. PPG sensors placed on the wrist or fingertip measure blood flow and oxygen saturation, while thermal sensors detect body heat at peripheral sites. Due to their minimal thickness and high flexibility, these sensors conform well to highly curved or mobile areas such as the elbow, knee, and shoulder, maintaining stable signal monitoring during movement. At joints, sensors can monitor gait, joint motion, or speech vibration (e.g., from the neck), supporting applications in rehabilitation and silent communication. Ultrathin wearable systems exploit material and structural innovations, including ultraflexible substrates, porous and nanofiber architectures for breathability, electronic tattoo (e-tattoo) platforms for imperceptible integration, and smart adhesives that offer both strong attachment and painless detachment (Figure 1d).^8^

In addition, many of these sensors also incorporate energy harvesting modules, enabling self-powered operation. Piezoelectric and triboelectric harvester elements can generate microwatts to milliwatts of power from high-strain areas like the knee or elbow. Smaller energy sources, such as body heat or shoulder motion, can also be harnessed using thermoelectric or hybrid systems to power low-consumption sensors or communication modules.^9^

While several excellent reviews have surveyed the landscape of ultrathin wearable sensors, they often focus on fabrication technologies^10^ or broad material and mechanism categories.^11^ In contrast, this review offers a distinct, bioinspired perspective on ultrathin sensors that focuses on the establishment of a skin-centric general framework. We begin by systematically analyzing the intrinsic properties of human skin, such as mechanical, electrical, and optical. We then use these properties as a guiding principle to critically evaluate the corresponding material choices, structural designs, and sensing mechanisms required to achieve high-fidelity biointerfacing. This approach allows us to bridge the gap between skin physiology and materials engineering, providing a targeted overview of how recent advances in synthesis, fabrication and integration directly address the challenges posed by the complex skin topography and mechanics. Finally, we summarize the potential for a broad spectrum of current and prospective applications and conclude with a discussion on the challenges and future outlook through this specific lens, aiming to provide a clear logic roadmap for the rational design of next-generation, truly skin-integrated devices.

## 2 Properties of Human Skin and Sensors, and Their Interfaces

As is known, live human skin is soft, stretchable, breathable, slightly conductive, and partially transparent (Figure 2). Skin provides a natural interface for sensing various internal and external stimuli. By emulating and leveraging skin-like features, wearable sensors can achieve conformal contact, stable signal acquisition, and user comfort. In the following sections, we consider how these intrinsic properties of skin guide the design of ultrathin, high-performance sensors, along with representative examples that demonstrate their functional advantages in real-world applications.

**Figure 2:**
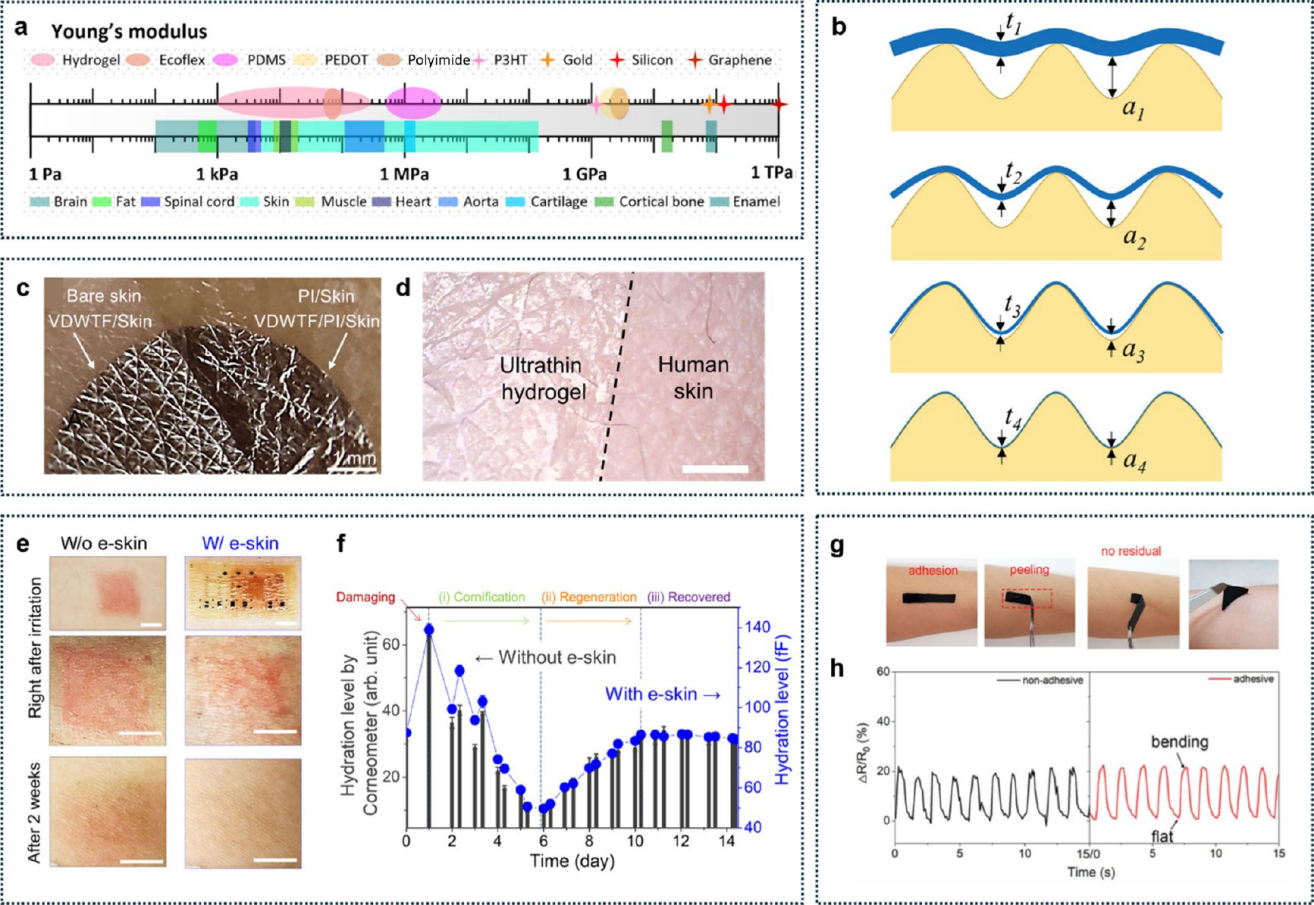
Summary of mechanical properties of human skin and skin-electronics interfaces. (a) Young’s moduli of representative man-made materials (top) and tissues (bottom). Reproduced with permission from ref 12. Copyright 2024 Elsevier. (b) Reducing film thickness (*t*_1_ > *t*_2_ > *t*_3_ > *t*_4_) improves its conformal contact by reducing the gap (*a*_1_ > *a*_2_ > *a*_3_ > *a*_4_) between the film and the substrate. Reproduced with permission from ref 14. Copyright 2019 Wiley-VCH. (c) Photograph of the freestanding van der Waals thin films (VDWTF) on a replica of human skin (left) and the VDWTF supported by a 1.6 mm-thick polyimide substrate on a replica of human skin (right). Reproduced with permission from ref 15. Copyright 2022 The American Association for the Advancement of Science. (d) Microscope image of the ultrathin hydrogel seamlessly attached to the human skin. Scale bar, 1 cm. Reproduced from ref 16. Available under a CC-BY 40 license. Copyright 2024 Zhang et al. (e) Skin regeneration monitoring for 2 weeks. Photographs of the inflamed skin region right after erythema and after 2 weeks, showing that perforated e-skin has a negligible adverse effect on skin regeneration. Scale bars, 5 mm. (f) Hydration level of the inflamed skin as a function of e-skin lamination days. Skin hydration level was monitored by a hydration sensor in the e-skin and simultaneously compared with a conventional hydration analyzer (Corneometer CM 825). Reproduced from ref 17. Available under a CC-BY 40 license. Copyright 2021 Yeon et al. (g) Demonstration of the adhesion between a strain sensor and human skin. The adhesive layer was water-dispersive polyurethane 30 wt %. No residual was left on the skin after the sensor was peeled off. (h) Signals from nonadhesive and adhesive sensors when the finger is bent down. Reproduced with permission from ref 18. Copyright 2021 Wiley-VCH.

### 2.1 Mechanical Properties of Human Skin and Skin-Electronics Interfaces

Human skin is exceptionally soft, elastic, and viscoelastic, with mechanical properties that vary across anatomical regions and layers. As shown in Figure 2a, the effective Young’s modulus of hydrated skin ranges broadly from 10 kPa to 1 MPa^12,13^ similar for common tissues such as fat, muscle, and heart. In contrast, elements of conventional electronic materials such as polyimide (PI) (∼2–5 GPa), gold (∼80 GPa), silicon (∼100–200 GPa), and graphene (up to ∼1 TPa) are many orders of magnitude stiffer. This disruptive mechanical mismatch can severely limit the ability of sensors to form robust contact with the skin, often leading to delamination and poor signal quality due to unstable mechanical contact.

To bridge this mechanical interfacial mismatch, skin-wearable sensors can be engineered to exhibit mechanical compliance close to that of the skin. One effective approach involves selecting intrinsically soft materials, such as hydrogels, Ecoflex, or polydimethylsiloxane (PDMS), which fall within the same modulus range as skin, below a few MPa. These elastomeric materials can deform synchronously with the skin, reducing interfacial stress. However, in cases where stiffer materials such as polyimide and parylene are required for their electronic or structural needs, the total thickness of the device can be minimized to reduce bending stiffness dramatically. Because bending rigidity scales with the cube of thickness, even modest thickness reductions can lead to substantial improvements in bending flexibility. Moreover, structural strategies such as incorporating serpentine, fractal, or mesh geometries further improve stretchability and reduce the effective resistance of the system.^19–21^ These designs allow the sensors to accommodate large deformations such as the ∼30% strain that can occur during joint flexion or facial expression without detachment or mechanical failure. By closely matching both the elastic modulus and mechanical dynamics of skin, ultrathin sensors can form stable, seamless contacts that maintain comfort and signal fidelity during natural movement.

Furthermore, human skin is not a flat surface but possesses rather a complex, microscopically textured topography of grooves, ridges (such as fingerprints), sweat pores, and hair follicles. Even surface areas that appear smooth contain undulating microstructures in the epidermis. These surface irregularities pose a significant challenge for achieving complete contact with wearable electronic devices. When sensors are relatively thick or rigid, they tend to bridge over skin asperities, leaving random microscopic air gaps (Figure 2b).^14^ Such gaps not only compromise mechanical adhesion but also increase contact impedance, degrading the quality of electrophysiological signal acquisition.

To overcome this issue, sensor designs increasingly rely on ultrathin and highly compliant structures that can mold seamlessly onto the skin’s microscopic topography. For instance, reducing the film thickness from *t*_1_ to *t*_4_ minimizes the gap (*a*_1_ to *a*_4_) between the sensor and the skin, allowing for near-perfect conformal contact (Figure 2b).

When the sensor thickness is reduced to the scale of a few micrometers—or ideally, hundreds of nanometers—van der Waals interactions and capillary forces can be sufficient to generate intimate adhesive contact without the need for extra adhesives (Figure 2c,d)^15,16,22^ Figure 2c shows that a rigid, high-modulus thin-film sensor constructed on bare skin can make more conformable contact compared to using a PI substrate (1.6 μm thick). However, it is important to note that achieving robust and reliable adhesion under diverse, real-world conditions—such as on hairy, sweating, or dynamically moving skin—remains a significant challenge. The long-term stability of such adhesive-free interfaces, particularly under repeated attachment and detachment cycles, is an area of ongoing research that requires further investigation to ensure practical viability.^23,24^ In contrast, Figure 2d illustrates a soft hydrogel-based film with a thickness of 10 μm, effectively adhering conformally to the skin. Moreover, a critical requirement for these sensors is breathability, as human skin continuously releases sweat. Thus, ultrathin sensors are often designed to be either inherently permeable or engineered with porous architecture, which facilitates moisture evaporation.

Preventing delamination is critical for reliable sensor operation and avoiding malfunctions. As demonstrated in Figure 2e–f, an ultrathin device composed of gold (Au), PI, and PDMS with an auxetic pattern showed no adverse effect on skin recovery, even when applied to inflamed tissue for 2 weeks.^17^ Skin hydration and temperature levels were maintained at physiologically normal ranges, comparable to untreated skin, supporting the device’s biocompatibility and prolonged use without compromising skin health. Figure 2g illustrates a skin-conformable device incorporating water-dispersible polyurethane, facilitating robust contact and leaving no residue upon removal.^18^ Figure 2h highlights the significance of an adhesive contact in achieving stable and consistent signal recordings during finger bending tests, clearly showing reduced noise and improved signal integrity.^18^

### 2.2 Electrical Properties of Human Skin

To clarify the general framework of this discussion, it is important to first distinguish the skin’s intrinsic electrical properties from the specific mechanisms that utilize them. The skin’s fundamental electrical property is its complex, frequency-dependent bioimpedance. This phenomenon arises from its multilayered structure, where the dry stratum corneum acts as a dielectric component (part of a capacitor) and the hydrated inner layers function as a conductor part. Sensing modalities, such as those for acquiring ECG and EMG, are corresponding mechanisms designed to exploit this conductive pathway, measuring surface biopotentials that propagate from underlying tissues. Thus, the skin’s electrical properties serve as the physical medium through which these physiological signals can be accessed.

For ultrathin, skin-conformable sensors designed to collect biosignals effectively, matching the impedance of these devices to that of human skin is critical to minimize signal attenuation and noise interference. Recent advancements in ultrathin sensor technologies emphasize the development of impedance-matched devices. Researchers typically adopt two primary approaches for creating skin-conformable electrodes: utilizing gel electrodes to adapt conformably to skin wrinkles or fabricating thin-film nanomembrane electrodes to reduce contact resistance.

Electrical impedance spectroscopy with noninvasive probes is commonly employed to measure the electrical current of human skin at varying frequencies (Figure 3a).^25,26^ At lower frequencies, impedance predominantly reflects the resistive properties of the extracellular environment. Conversely, at higher frequencies, impedance arises from both the resistive characteristics of intra- and extracellular environments and the capacitive attributes of cell membranes. Figure 3b,c present calculated resistivity and relative permittivity values of human skin, respectively.^25^ Specifically, the resistivity of the stratum corneum, a layer within the epidermis, is approximately 10^5^ Ω·m at frequencies around 10 Hz.

**Figure 3:**
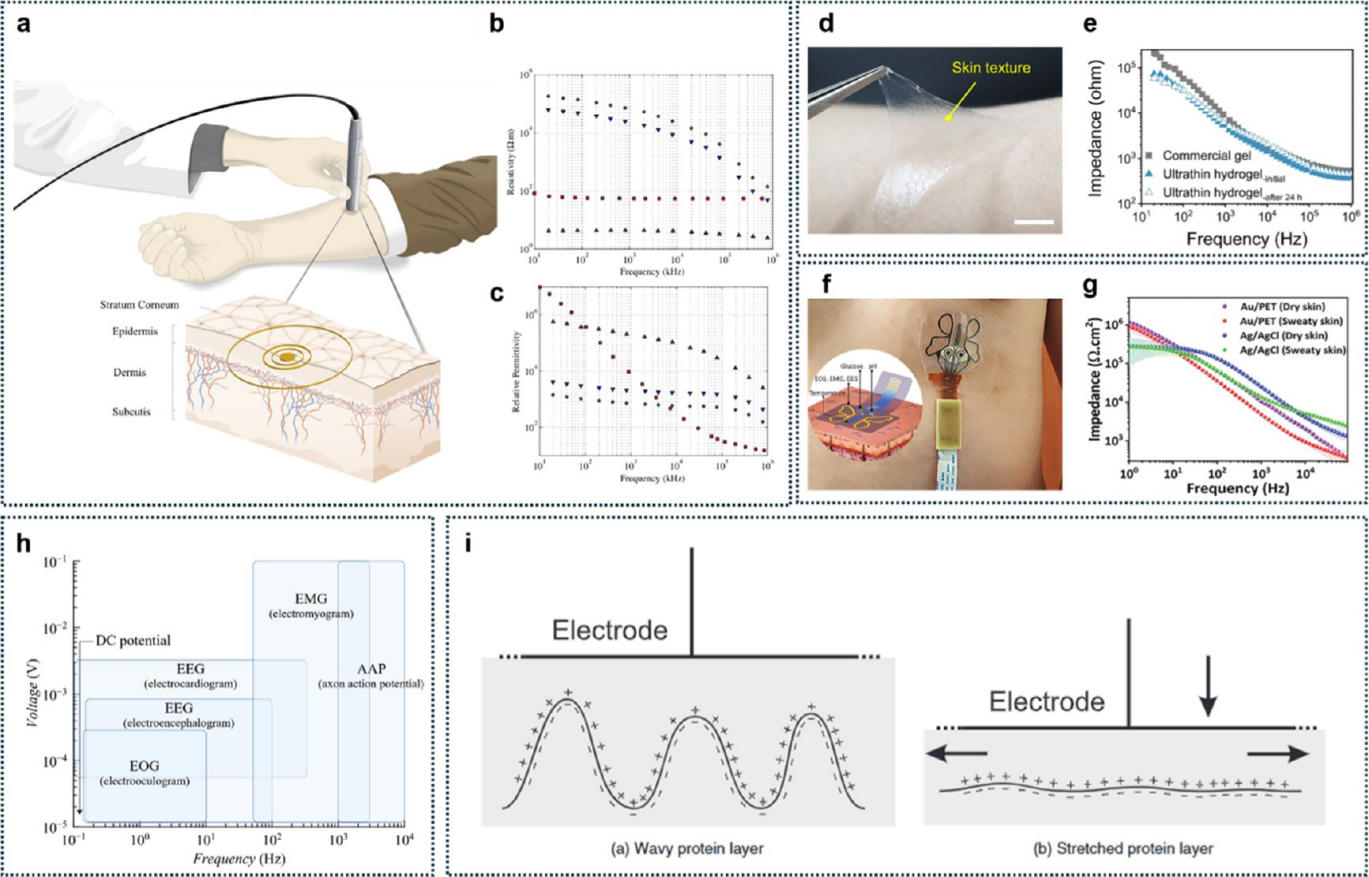
Electrical properties of human skin. (a) Schematic overview of the noninvasive probe applied to the forearm and a detailed image of the layers of the skin. (b) Average resistivities of stratum corneum, adjusted stratum corneum, viable skin, and adipose tissue. (c) Average relative permittivity of stratum corneum, adjusted stratum corneum, viable skin, and adipose tissue. Reproduced with permission from ref 25. Copyright 2010 IOP Publishing. (d) Photograph of the peeling-off of an ultrathin hydrogel from the human skin, showing strong adhesion and elasticity. Scale bar = 1 cm. (e) Skin-electrode contact impedance analysis of commercial gels and ultrathin hydrogels. Reproduced from ref 16. Available under a CC-BY 40 license. Copyright 2024 Zhang et al. (f) Magnified view of the patch attached to the chest, inset—the multiplex electrochemical-physical flexible sensor array. (g) Observation of skin-contact impedances of the fabricated Au/PET and conventional Ag/AgCl electrodes for dry skin and sweaty skin. Reproduced with permission from ref 27. Copyright 2022 Wiley-VCH. (h) Frequency spectrum of various biopotential and mechanical signals. Reproduced from ref 28. Available under a CC-BY 40 license. Copyright 2020 Kledrowetz et al. (i) Schematic representation of layers of charged proteins in the skin; left—in tissue with wavy protein layers, the Donnan potentials may cancel out, right—The Donnan potentials align when the tissue is compressed, and the measured potential increases. Reproduced with permission from ref 29. Copyright 2003 Taylor & Francis.

As an example, Figure 3d illustrates a skin-conformable gel electrode consisting of flexible polyurethane (PU) nanomesh dip-coated in an ionic cross-linked hydrogel solution.^16^ The resultant gel electrode, only 10 μm thick, demonstrated an impedance of 31.3 kΩ at 100 Hz, significantly lower than commercial gel electrodes (1 mm thick) with impedances around 57.5 kΩ at the same frequency. Importantly, this ultrathin hydrogel electrode maintained stable impedance values even after 24 h of continuous operation (Figure 3e).^16^

Additionally, Figure 3f showcases an epidermal patch device based on thin-film technology, designed to capture various electrophysiological signals directly from human skin through conformable adhesion.^27^ This device incorporates two distinct electrodes: silver (Ag)/AgCl and Au/polyethylene terephthalate (PET). Initial skin-contact impedance measurements for these electrodes showed 250.7 and 310.5 kΩ·cm^2^, respectively. The larger surface area of the Au/PET electrode contributed to its superior electrochemical performance, as evidenced by reduced skin contact impedance. Specifically, at 10 Hz, the mean impedance of Au/PET and Ag/AgCl electrodes measured 222.6 and 210.4 kΩ·cm^2^, respectively (Figure 3g).^27^ This notable impedance reduction results from sweat acting as an electrolyte with the presence of ions such as Na^+^, Cl^–^, and K^+^. Thus, impedance changes induced by sweating must be accounted for in the design and signal processing for high acquisition accuracy.

Most biological signals occur at low frequencies and possess amplitudes at the microvolt level, necessitating advanced signal processing and tailored design considerations to achieve optimal selective gain and bandwidth.^30–32^ To this end, Figure 3h details the frequency characteristics of various biomedical signals.^28^ Lastly, Figure 3i schematically depicts the Donnan potential arising from skin.^29^ Not only the human skin’s different parts have different electric properties,^33^ when skin tissues undergo mechanical deformation (e.g., pressing), the stretched wavy protein layers result in increased potentials compared to their equilibrium states. To avoid misinterpretation of signals stemming from such mechanical artifacts, employing accurately engineered skin-conformable devices is essential for precise signal acquisition and interpretation.

### 2.3 Optical Properties of Human Skin

Next, the skin’s primary optical appearance is characterized by its wavelength-dependent absorption (μ_a_) and scattering (μ_s_) coefficients. These properties are determined by the concentration of various chromophores, most notably melanin, and oxygenated/deoxygenated hemoglobin. Therefore, techniques like photoplethysmography (PPG) are sensing mechanisms that leverage this principle. PPG sensors emit light at specific wavelengths to probe these intrinsic differences in hemoglobin’s light absorption, thereby enabling the monitoring of physiological state.

In general, healthcare monitoring and treatment using optical methods offer significant advantages due to their noninvasive nature and ability for continuous monitoring.^34–37^ Precise diagnostics using optical sensors depend on understanding the penetration depths of light at various electromagnetic (EM) wavelengths (Figure 4a).^38–40^

**Figure 4:**
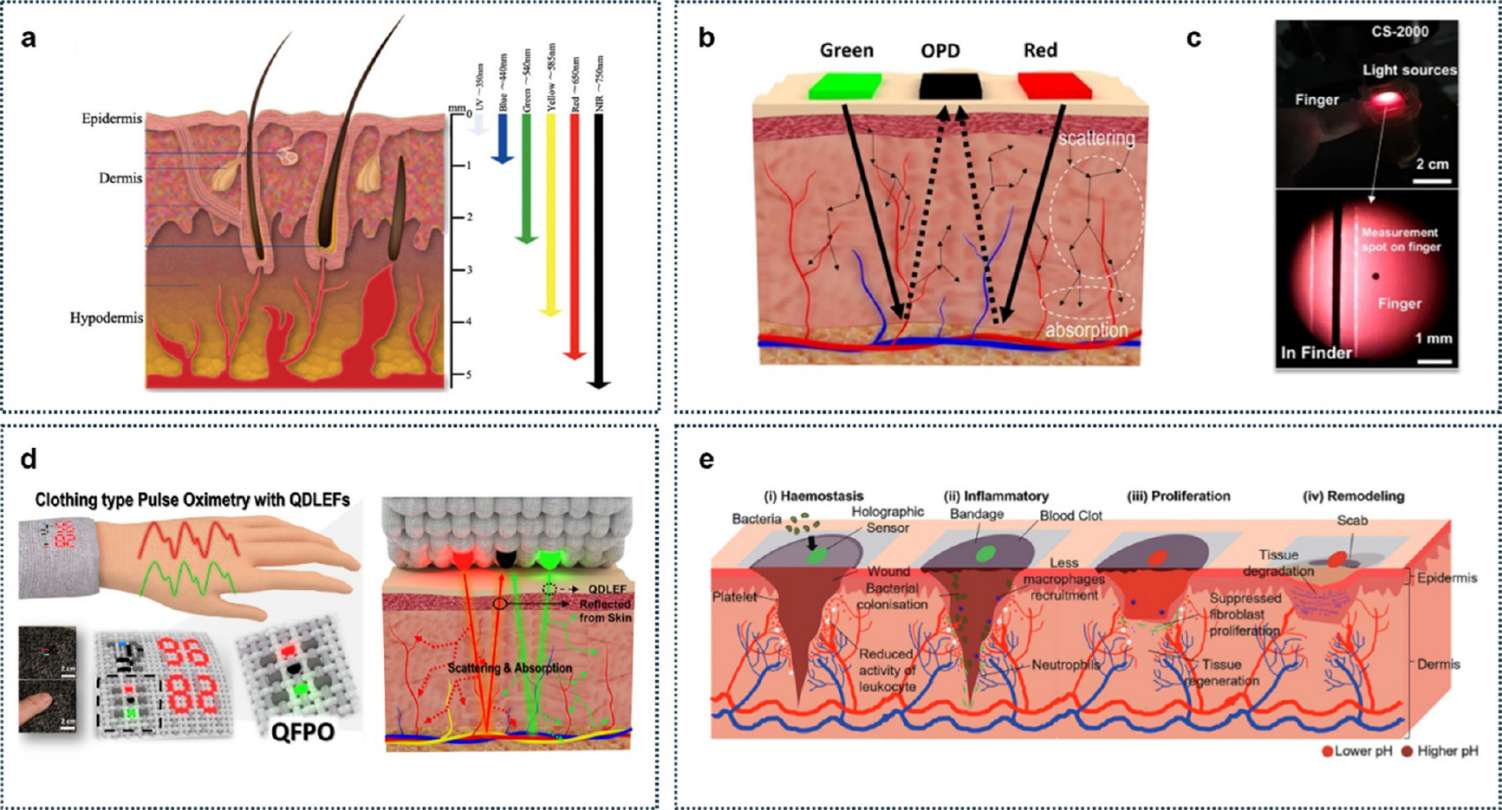
Optical properties of human skin. (a) Penetration depth of UV, visible light, and NIR radiation in human skin. UV radiation has the shortest wavelength and is not capable of penetrating deeper than the epidermis, while NIR can target deep tissue in the hypodermis and blood vessels. Reproduced from ref 38. Available under a CC-BY 40 license. Copyright 2022 Sutterby et al. (b) Schematic illustration of the reflected light path based on the fiber-based quantum dot pulse oximetry due to the scattering and absorption phenomena in human skin. (c) Photo of the experimental environment and method for measuring the reflected light characteristics from human skin. Reproduced from ref 42. Available under a CC-BY 40 license. Copyright 2023 Lee et al. (d) Schematic illustration of the concept and photos of the quantum-dot light-emitting fiber pulse oximetry-based clothing type pulse oximetry using quantum-dot light emitting fiber (top and bottom inset, scale bar: 2 cm). Reproduced from ref 43. Copyright 2024 American Chemical Society. (e) Wound healing monitoring via holographic sensors with a broad viewing angle. The impact of alkaline pH of wound exudate in four wound healing phases (i) hemostasis phase. (ii) Inflammatory phase. (iii) Proliferation phase. (iv) Remodeling phase. Reproduced with permission from ref 44. Copyright 2024 Wiley-VCH.

Among the most popular optical methods, PPG for health monitoring is used to measure the oxygen saturation levels in hemoglobin.^41^ Figure 4b illustrates the underlying mechanism of PPG, highlighting how different wavelengths result in varied scattering and light absorption characteristics within the skin, detectable via organic photodiodes (OPDs).^42^

Figure 4c demonstrates an experimental setup for measuring reflected light characteristics from human skin.^31^ The selective sensitivity arises from the various skin layers—epidermis, dermis, lipids, and muscle—each containing distinctive structures such as mitochondria, collagen fibers, lysosomes, and lipid–water interfaces. Pulse oximetry^43^ analyzes light penetration and reflection to a photodiode (Figure 4d).^41,45,46^ Quantum-dot (QD) light-emitting fiber pulse oximetry which is shown in the figure enables detection from the skin simply by wearing clothes having QD light emitting fiber. Variations in blood volume with each cardiac cycle allow detection of reflected light absorption differences between deoxygenated hemoglobin and oxygenated hemoglobin, enabling accurate determination of oxygen saturation.

Figure 4e illustrates an innovative wound healing and monitoring bandage employing optical methods.^44^ As the bandage absorbs moisture during healing, it swells, altering its interference patterns that allow for monitoring wound healing progress through holographic sensing. This versatile technology holds the potential for sensing biomarkers, such as glucose or magnesium ions.

### 2.4 Biocompatibility Requirements

Thorough investigation and development of biocompatibility for skin-conformable electronics during prolonged interactions between human skin and electronic devices is essential.^47^ Skin inflammation might occur at skin-electronics interfaces (Figure 5a).^48^ Conductive inks can interact adversely with biological tissues, potentially causing cellular apoptosis, skin inflammation, and irritation with prolonged use. Detection of another inflammation mechanism is demonstrated in Figure 5b,^49^ where interactions between epidermal cells and encapsulated ink particles, including carbon and Ag/AgCl, were studied to determine the toxicological pathway of particle leakage.

**Figure 5:**
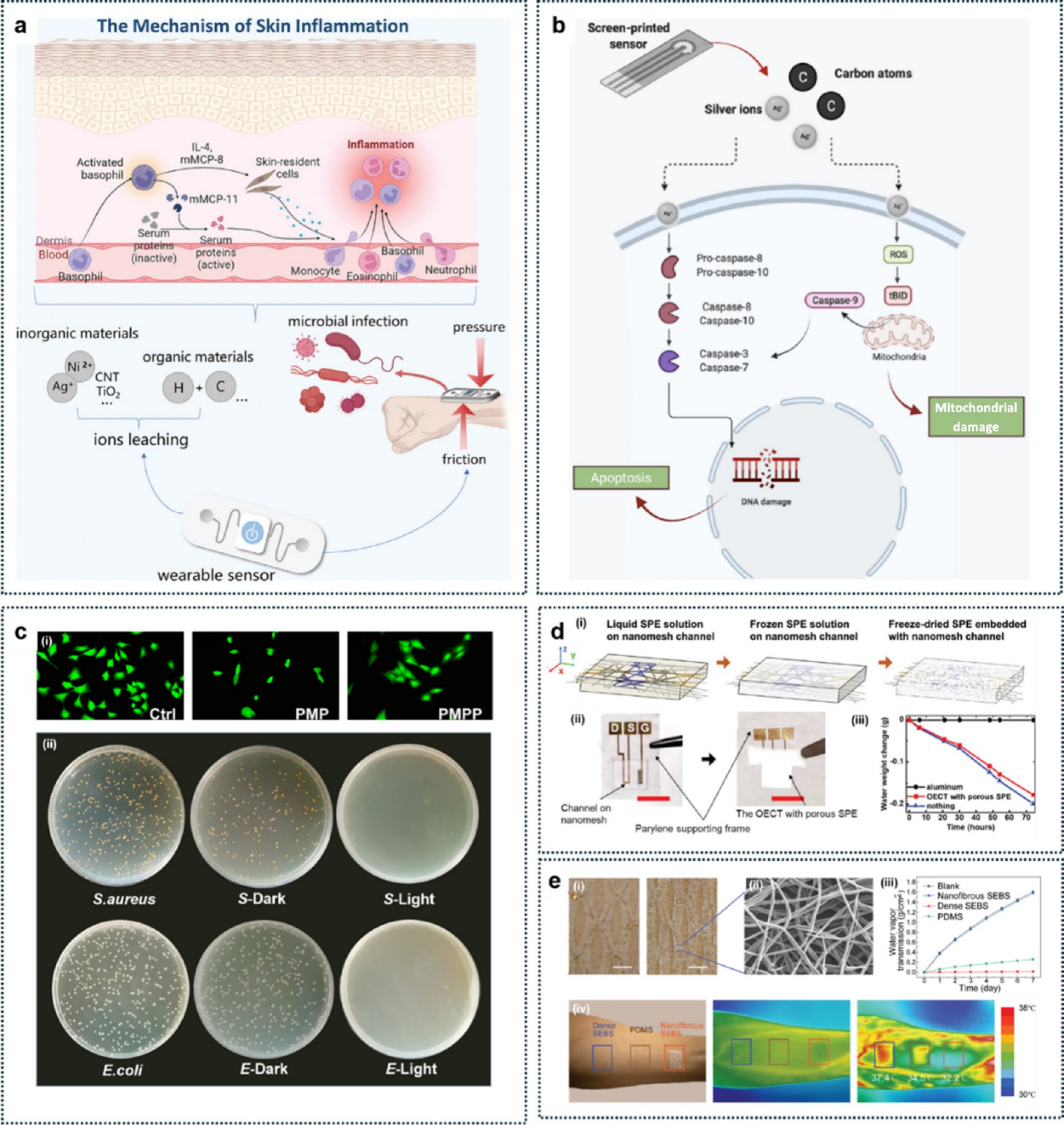
Biocompatibility requirements. (a) The mechanism diagram of wearable devices causing skin inflammation. Reproduced with permission from ref 48. Copyright 2024 Wiley-VCH. (b) Schematic of wearable sensors interacting and eliciting a biological response. Reproduced from ref 49. Available under a CC-BY 40 license. Copyright 2022 Bhushan et al. (c) Cell relative viability of mouse cells (NIH-3T3) treated with different samples. (i) Typical fluorescence microscopy image of NIH-3T3 after incubation for 1 day on the substrate of Control, PMP, and PMPP. (ii) Digital images of agar plates used for the growth of *E. coli* and *S. aureus* exposed to PMPP film for 20 min under conditions with or without near-IR (808 nm) laser irradiation. SEM images of the untreated. Reproduced from ref 50. Available under a CC-BY 40 license. Copyright 2022 Wang et al. (d) (i) Schematic to show the process of embedding the porous SPE into the nanomesh organic electrochemical transistor (OECT). A fabricated nanomesh OECT using the parylene film as the supporting frame; (ii) left—the center parts are freestanding nanomesh (scale bar: 5 mm). Right—the porous SPE embedded nanomesh OECT (scale bar: 5 mm). (iii) Water vapor evaporation results of the fabricated porous SPE embedded nanomesh OECT compared with those of a continuous film (aluminum foil) and an open environment. Reproduced with permission from ref 54. Copyright 2024 Wiley-VCH. (e) (i) Photographs showing serpentine electronics on top of the nanofibrous substrate (left) and in conformal contact (right) with the skin surface. Scale bar: 10 mm. (ii) A scanning electron microscopy (SEM) image showing the permeable nanofibrous structure of a breathable and conformable electronic membrane (BCEM). Scale bar: 5 μm. (iii) Water vapor transmission as a function of time for dense SEBS film, nanofibrous SEBS membrane, PDMS film, and blank control. (iv) A photograph (left) showing three different samples laminated on the forearm, and infrared thermal images before (middle) and after (right) the running exercise, demonstrating the breathability of a nanofibrous SEBS as compared with dense SEBS and PDMS film. Reproduced with permission from ref 55. Copyright 2022 Wiley-VCH.

Cell viability assays are among the most prevalent approaches to confirm the safety of the proposed materials. For sensory devices that directly contact or penetrate human skin, the cell viability test is mandatory to show that the device is safe to use.^50^ Figure 5c shows the results of cell viability tests and photothermal antibacterial responses for a polyurethane–MXene–PDMS–polydopamine (PMPP) composite.^51^ Compared to a polyurethane-MXene–PDMS structure, the PMPP composite demonstrated higher cell viability. Furthermore, exposure to IR laser irradiation at 0.27 W cm^–2^ effectively eliminated bacterial strains such as *Staphylococcus aureus* and *Escherichia coli*.

Another method for evaluating biocompatibility is assessing breathability via water evaporation rates.^52,53^ Figure 5d presents a micromesh-structured device fabricated through freeze-drying methods using solid-state polymer electrolytes (SPE).^54^ This porous SPE demonstrated excellent breathability compared to nonporous aluminum substrates, which exhibited no breathability. Such porous structures, by allowing unhindered water vapor transmission, reduce the likelihood of skin inflammation.

Figure 5e highlights a combination of electrospinning and laser-cutting methods for the fabrication of breathable skin-conformable sensors.^55^ Introducing kirigami structures through laser cutting not only enhanced device breathability compared to dense PDMS or styrene–ethylene–butadiene–styrene (SEBS) films, but also improved mechanical adaptability to the dynamic skin environment. Breathability was quantitatively assessed by measuring the water-vapor transmission rate, determined from the weight loss of water-filled containers sealed with the fabricated membranes. In addition, the open kirigami patterns effectively reduced mechanical constraint and enabled conformal deformation under stretching, bending, and twisting motions of the skin. This structural strategy minimizes delamination and mechanical fatigue, thereby supporting long-term usability under repeated attachment/detachment cycles and in conditions involving sweat and continuous body movements.

## 3 Material Selection and Structures for Wearable Soft Sensors

### 3.1 Material Selection and Structural Design

The sensing materials must exhibit not only the required electrical or electrochemical properties tailored to their function but also mechanical characteristics, including low Young’s modulus and high fracture strain, biocompatibility, and processability into uniform ultrathin films.^56,57^ To ensure the operational stability of these devices under mechanical deformation, carefully engineered structural designs are essential. Strategies such as wrinkled surfaces,^58^ porous structures,^59^ or serpentine interconnects^60^ have proven to be effective. Notably, ultrathin form factors can greatly enhance conformability to deformable biological interfaces. Furthermore, the overall performance can be enhanced through optimization of surface patterning and film thickness, aiming to maximize interfacial effects or improve transduction efficiency for high mechano-electric conversion.

#### 3.1.1 Planar Sensory Structures

Minimizing the overall thickness and weight of sensors enhances wearability and reduces mechanical mismatch with biological tissues at the interface, thereby alleviating interfacial stress.^61^ To fully leverage the advantages of these ultrathin substrates, the active sensing components must also meet specific requirements. These functional materials should form uniform thin films, excellent flexibility, and reliable electrical and mechanical properties to maintain high performance of ultrathin substrate platforms.^62,63^

Among popular materials choices, graphene possesses a range of highly desirable properties, including chemical stability, mechanical flexibility and durability, electrical conductivity, and biocompatibility, making it a promising material for developing flexible sensors.^64^ However, the reliable transfer of high-quality graphene films onto soft substrates remains a major challenge in wearable device fabrication. To address these issues, researchers have explored the self-assembly of graphene in combination with substrate surface treatments.

To this end, He et al. demonstrated the fabrication of large-area stretchable graphene devices on ultrathin PDMS substrates by integrating high-quality graphene nanosheets with plasma-induced wrinkles (Figure 6a).^65^ This approach produced wafer-scale, intrinsically stretchable graphene-elastomer composites with nanoscale wrinkling, enabling the formation of conformal arrays. By tuning the wrinkle morphology, achieved by transferring graphene onto plasma-treated PDMS, the resulting devices exhibited stable electrical performance under strains up to 80% and in some cases up to 100% when fabricated on 50% prestrain. This fabrication strategy employs photolithography and shadow mask techniques without harsh processes, simplifying production and minimizing substrate damage. The stretchable pressure sensor array demonstrated promising triboelectric nanogenerator (TENG) performances of 150 V of open circuit voltage and 2 μA of short circuit current even under 100% strain without signal degradation. The engineered wrinkle structure ensures strain-insensitive behavior, maintaining consistent sensor response under deformation. This approach offers a promising strategy for graphene-based stretchable soft electronic devices for applications like wearable sensors.

**Figure 6:**
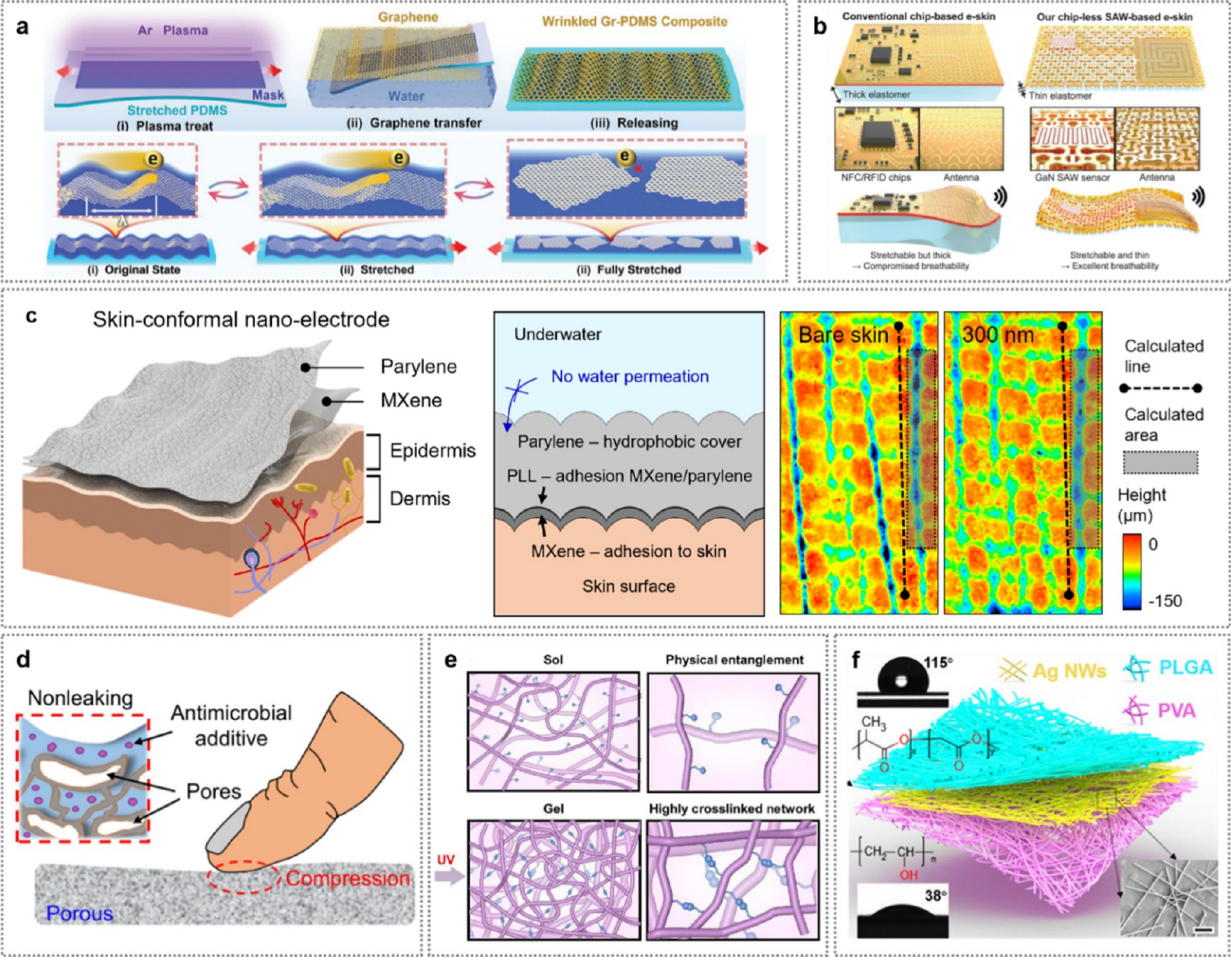
Planar and porous structure for ultrathin soft devices. (a) Intrinsically stretchable wrinkled graphene-elastomer composite for a strain-insensitive tactile sensor. Reproduced with permission from ref 65. Copyright 2022 Wiley-VCH. (b) Ultrathin single-crystalline piezoelectric GaN membrane-based chipless wireless surface acoustic wave sensor. Reproduced with permission from ref 67. Copyright 2022 The American Association for the Advancement of Science. (c) Robust skin-conformal nanoelectrodes for sustainable health and performance monitoring in challenging environments. Reproduced from ref 68. Copyright 2025 American Chemical Society. (d) Porous liquid metal-elastomer composite with high leakage resistance for bioelectronic conductors. Reproduced from ref 69. Available under a CC-BY 40 license. Copyright 2023 Xu et al. (e) Polyimide aerogel fibers for ultrathin thermal management fabrics. Reproduced from ref 70. Available under a CC-BY 40 license. Copyright 2023 Xue et al. (f) Multilayer interlaced nanofiber and three-dimensional hierarchical pores for breathable and self-powered electronic skin. Reproduced from ref 71. Available under a CC-BY 40 license. Copyright 2020 Peng et al.

As is known, conventional wireless e-skin sensory systems often employ rigid integrated circuit chips for wireless communication and signal processing, which compromises flexibility and leads to high power consumption. In contrast to these rigid IC-based systems, chipless LC resonator-based sensors have emerged as a potential alternative for wireless sensing without integrated circuits.^66^ However, their applications remained limited, mainly focusing on strain and pressure sensing. Therefore, to achieve ultrathin soft sensors, it is essential to develop chipless wireless E-skin technology that maintains flexibility while offering high sensitivity and power efficiency.

In this effort, Kim et al. introduced ultrathin, substrate-free E-skins by assembling freestanding single-crystalline piezoelectric gallium nitride (GaN) membranes (Figure 6b).^67^ The electronic skin system includes a GaN surface acoustic wave (SAW) sensor for detecting external stimuli, a flexible PDMS patch to support the sensor and ensure skin adhesion, stretchable electrodes and interconnects for electrical connection and communication with a reader, and an antenna for wireless data transfer. The fabrication process involves remote homoepitaxy to grow high-quality single-crystalline GaN thin films on 2D material-coated GaN substrates, followed by a 2D material-based layer transfer process to exfoliate and transfer the GaN film onto a flexible substrate. This design enables the electronic skin to achieve performance metrics like 0.048% minimum detectable strain, 10.06 A W^–1^ ultraviolet (UV) light responsivity at 15.9 mW cm^–2^, enhanced flexibility, and long-term wearability. This system offers continuous pulse measurement for cardiovascular health monitoring and a sweat sodium ion concentration analysis for assessing health conditions.

High conformability to the skin allows for a “second skin-like” wearing experience and records high-quality biosensor signals over an extended time in challenging environments. Kim et al. reported a trilayer planar ultrathin electrode consisting of a hydrophilic MXene conductive film, a poly-l-lysine molecular adhesion layer, and an ultrathin hydrophobic parylene substrate (∼300 nm thickness) (Figure 6c).^68^ Ultrathin amphiphilic nanoelectrodes using water-assisted capillary-driven flow offer significantly enhanced physical conformability and resilient electrical properties, making them a robust platform for low-motion artifact and water-resistant electrophysiological monitoring. The unique design of dual wettability, incorporating a MXene conductor for enhanced skin adhesion and a hydrophobic cross-linked supporting polymer layer with hydrophobic properties, ensured strong contact with the skin, even during intense motions in air and underwater. Structural analysis demonstrated that a nanoelectrode provides strong, highly adherent physical conformal contact with the skin, effectively adapting to the intricate, complex textures of human skin.

These ultrathin, skin-conformal nanoelectrodes combine exceptional durability, low skin impedance, and water resistance, enabling stable, high-fidelity ECG and EMG monitoring in challenging conditions, including saunas and underwater. Their submicron, conformal design ensures reliable signal acquisition with 3–5× improved signal-to-noise and minimal motion artifacts (by 30–60%), over traditional gel or thicker electrodes. Supported by extensive structural, mechanical, and environmental validations, these electrodes present a robust, long-term platform for continuous bioelectronic monitoring.

#### 3.1.2 Porous Sensory Structures

Compared to planar materials, porous structures offer variable mechanical properties combined with a large specific surface area, which improves the sensitivity.^72^ The high compressibility and flexibility of porous structures enable them to respond sensitively to subtle mechanical stimuli, thereby improving the performance of pressure and strain sensors.^73^ Porous structures can be achieved through various methods, including sponge-like foams,^74^ 3D-printed architectures,^75^ and textile-based structures.^76,77^ This structural versatility enables their integration with a wide range of materials, making them a promising platform for developing multifunctional and high-performance soft sensors.

As an example, Xu et al. developed porous composites composed of eutectic gallium–indium (EGaIn), PU, and ε-polylysine (ε-PL) using a phase separation process, during which the liquid metal self-organizes into conductive pathways (Figure 6d).^69^ These porous composites showed superior resistance to leakage, outperforming their nonporous counterparts under compression. For instance, nonporous composites leaked approximately 0.22 g cm^–3^ at 400% strain, whereas porous composites exhibited negligible leakage. Moreover, the porous composites showed a lower percolation threshold (∼0.07) compared to that of nonporous composites (0.39), indicating that less liquid metal was required to achieve similar electrical conductivity. They also exhibited high conductivity (∼10^6^ S m^–1^) and skin-like mechanical properties, with a modulus of ∼1.5 MPa, much lower than that of nonporous composites (∼16.7 MPa). In addition, these materials exhibited excellent breathability and strong antimicrobial and antiviral effects, achieving ∼97% inactivation of SARS-CoV-2. These characteristics make the porous composites highly suitable for next-generation bioelectronics and wearable devices. Their design minimizes discomfort and allows conformal contact with the skin, while breathability improves long-term wearability by preventing moisture buildup.

In another example, thermoregulating textiles and aerogels possess energy efficiency, are lightweight, porous, and ultrathin. However, the fabrication of aerogel fibers has been limited by long sol–gel reaction times and complex drying processes. For these materials, Xue et al. developed substrate-free soft textiles using cross-linked polyimide (CPI) aerogel fibers with unique porous structures (Figure 6e).^70^ Their scalable method combines UV-induced rapid gelation, wet spinning, and ambient pressure drying, enabling continuous fiber production within 7 h, far faster than conventional 48 h methods. The resulting CPI-100 aerogel fibers exhibit a high specific modulus of 390 kN m^–1^ kg^–1^ and can be woven into an ultrathin fabric just 0.7 mm thick. They offer thermal insulation comparable to down with a low thermal conductivity of 24.2 mW m^–1^ K^–1^ at −50 °C. The integration of CPI aerogel fibers with phase change materials has enabled thermally adaptive fabrics suitable for smart thermal regulation in high-temperature environments. This approach offers a scalable and cost-effective fabrication of high-performance, multifunctional aerogel fibers, demonstrating significant potential for personal thermal management.

To achieve a comfortable, safe, and eco-friendly wearable device, a breathable, biodegradable, and antibacterial design with an ultrathin profile and porous structure represents a significant advancement. To demonstrate this, Peng et al. developed a substrate-free e-skin using a multilayered nanofiber network (Figure 6f).^71^ This E-skin incorporates silver nanowires (AgNWs), polylactic-*co*-glycolic acid (PLGA), and poly­(vinyl alcohol) (PVA) to form a three-dimensional microto-nano hierarchical porous structure with high surface area, which is advantageous for contact electrification and pressure sensing. Furthermore, it incorporates numerous capillary channels that facilitate the transfer of heat and moisture. The multilayered nanofiber network enables high breathability, with an air permeability of approximately 120 mm s^–1^, significantly higher than that of commercial textiles, thereby facilitating natural skin respiration and effective sweat and heat dissipation. Notably, the antibacterial and biodegradable capabilities of the e-skin can be customized by adjusting the concentration of AgNWs, which inhibits the growth of bacteria on the skin, reducing the risk of infection. The developed multilayered nanofiber network can be utilized as a TENG-based electronic skin for real-time monitoring of physiological signals and diverse joint movements of the human body. It exhibits a maximum peak power density of 130 mW m^–2^ and a voltage response pressure sensitivity of 0.011 kPa^–1^. This work addresses key limitations of conventional materials and offers promising potential for practical wearable and medical devices.

#### 3.1.3 Hybrid Materials and Composite Structures for Sensory Systems

Hybrid nanocomposite materials that integrate stretchability and electrical conductivity have emerged as key materials for wearable electronics. These materials are particularly critical for biointegrated sensors that require both mechanical compliance and stable electrical performance under deformation. To this end, the ultrathin geometry facilitates conformal integration with irregular biological surfaces during motion.

A widely adopted strategy to achieve this involves embedding conductive fillers into thin elastomeric matrices to form hybrid architectures that couple softness with electrical functionality. For example, the combination of intrinsically elastic polymers with conductive fillers such as carbon-based materials,^78,79^ metal,^80–82^ or conductive polymers like traditional PEDOT:PSS,^83–85^ enables the fabrication of flexible and stretchable electrodes. However, hybrid systems often face challenges such as limited durability under repeated deformation. Addressing these issues requires nanoscale structural engineering and robust interfacial optimization to enhance both electrical and mechanical reliability in dynamically moving environments.

Enhancing electrical conductivity is a fundamental requirement for achieving stable signal acquisition in epidermal electronic devices. Hybrid composites employing different conductive fillers can synergistically improve charge transport, enabling high-performance electrodes. By using this approach, Zhao et al. developed an ultrathin (∼100 nm-thick) dry electrode by integrating PEDOT:PSS and graphene, thereby enhancing conductivity and stretchability (Figure 7a).^83^

**Figure 7:**
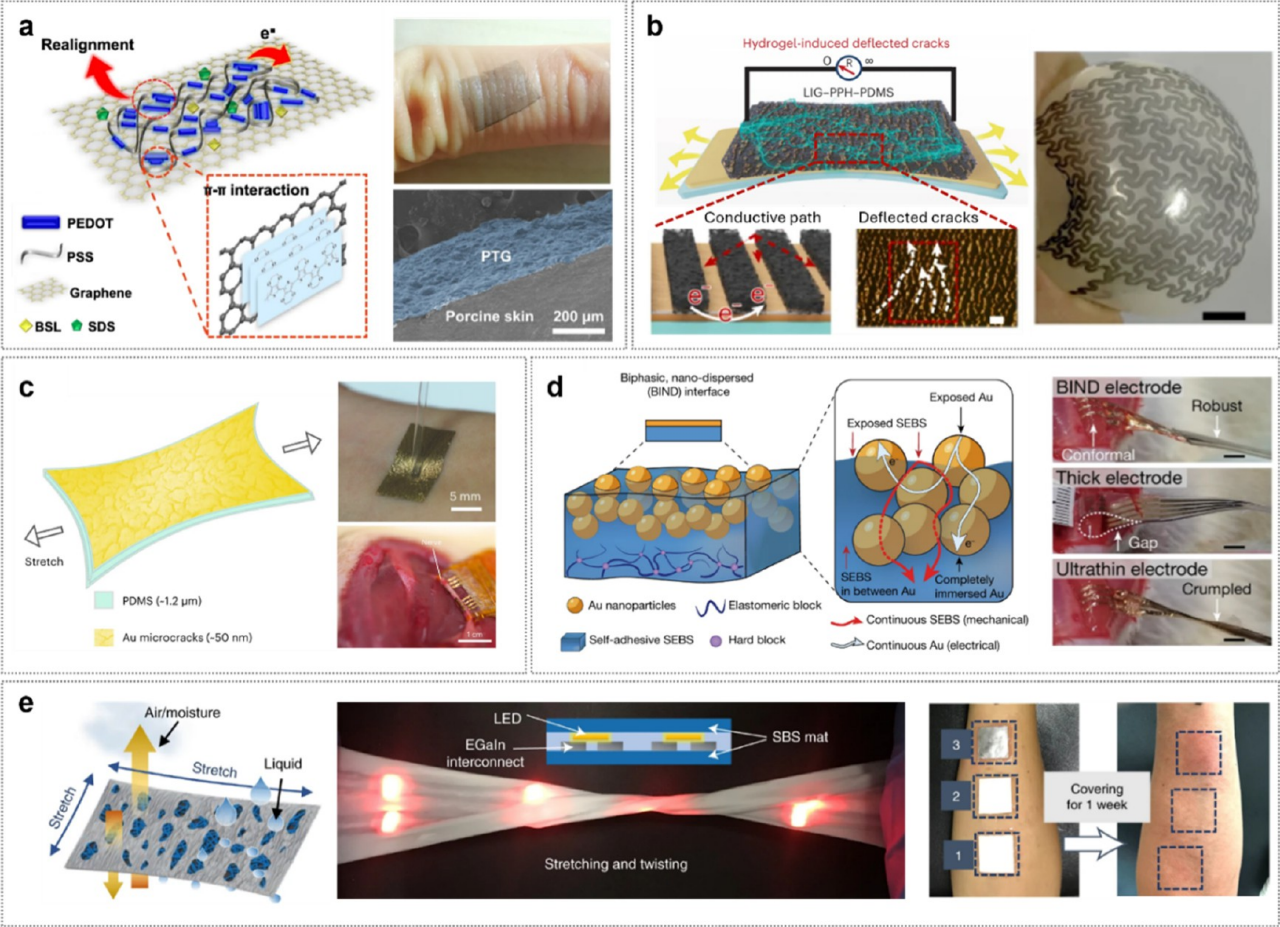
Hybrid materials and composite structures. (a) Synergetic enhancement in electrical conductivity via π–π interaction and charge delocalization between graphene and PEDOT:PSS (left). Photograph of an ultrathin sensor conformally integrated onto a finger, and a cross-sectional SEM image showing the ultrathin electrode attached to porcine skin (right). Reproduced from ref 83. Available under a CC-BY 40 license. Copyright 2021 Zhao et al. (b) Crack deflection within a LIG layer integrated with a PPH interlayer enables the formation of deflected conductive pathways, ensuring stable electrical performance (left). Photograph of a thin LIG–PPH–PDMS composite conformally adhered to a balloon surface (right). Reproduced with permission from ref 86. Copyright 2023 Springer Nature. (c) Stretchable PDMS–Au microcracked composite that maintains conductivity under strain (left). Photographs showing its application on human skin and a rat sciatic nerve (right). Reproduced with permission from ref 80. Copyright 2022 Springer Nature. (d) Hybrid nanodispersed interface composed of interpenetrating Au nanoparticles and SEBS domains, forming continuous electrical networks (left). Photograph showing conformal application onto the peroneus longus muscle (right). Reproduced with permission from ref 81. Copyright 2023 Springer Nature. (e) Permeable LMFM exhibiting high stretchability and biocompatibility (left). Photographs showing the results of a skin irritation test after 1 week of application (middle), and encapsulated composites with printed EGaIn interconnects powering LEDs under twisting deformation (right). Reproduced with permission from ref 87. Copyright 2021 Springer Nature.

It has been shown that while PEDOT:PSS material offers intrinsic conductivity and biocompatibility, its mechanical brittleness under strain limits its application in dynamic epidermal interfaces. In this hybrid configuration, π–π stacking interactions between PEDOT chains and graphene promote molecular alignment, while the extended π-electron enhances charge carrier mobility via delocalization. The hybrid electrode achieves high conductivity (∼4142 S cm^–1^), low sheet resistance (∼24 Ω sq^–1^), and optical transparency. Due to its nanoscale thickness, the electrode conforms intimately to complex skin surfaces. It enables high-fidelity monitoring of EMG, ECG, EOG, and long-term EEG signals, making it a promising platform for wearable bioelectronics.

On the other hand, hydrogel-based composites provide a skin-friendly interface with enhanced mechanical compliance and hydration. Ultrathin hydrogels are well-suited for biointerfaces due to their high stretchability and biocompatibility. To impart electrical conductivity to hydrogel matrices, a variety of conductive fillers have been explored, including conducting polymers PEDOT:PSS,^84,88^ liquid metals,^82^ and graphene.^86^ In a recent study, Lu et al. developed graphene-hydrogel hybrid bioelectronics by cryogenically transferring laser-induced graphene (LIG) onto an ultrathin (∼1.5 μm) hydrogel composed of PVA, phytic acid, and honey (PPH), followed by integration onto a 10 μm-thick PDMS layer (Figure 7b).^86^ LIG offers advantages such as simple patternability, transfer printing compatibility, and tunable surface chemistry. The low-temperature processing enhanced the interfacial bonding between defect-rich porous graphene and the crystallized water within the hydrogel. Acting as a stress-dissipating interfacial layer, the PPH hydrogel guided crack propagation along deflected paths, thereby preserving electrical conductivity under mechanical strain. As a result, the LIG–PPH–PDMS composite maintained stable conductivity under tensile strains up to ∼220%. The graphene-hydrogel-based wearable soft sensor system enables stable monitoring of diverse biosignals, including respiratory humidity and ECG.

Recent efforts to develop ultrathin, stretchable electrodes have focused on the integration of electric conductors with soft elastomers. Jiang et al. demonstrated a 1.3 μm-thick hybrid electrode by thermally evaporating a 50 nm gold layer onto a 1.2 μm PDMS film (Figure 7c).^80^ Thermal mismatch during deposition induces a controlled microcrack morphology in the ultrathin Au layer, which guides the formation of nonparallel cracks under mechanical strain. These features enable stretchability up to ∼300% while preserving electrical conductivity through connected Au microfragments. The low modulus and ultrathin geometry further support conformal contact and stable ECG signal acquisition during vigorous motion.

In a separate study, Jiang et al. also developed a hybrid nanodispersed interface by thermally evaporating Au or Ag nanoparticles onto the surface of a SEBS thermoplastic elastomer (Figure 7d).^81^ The resulting interpenetrating nanostructure penetrates ∼90 nm into the SEBS matrix, forming a hybrid interface with exposed metal domains for Ohmic contact and polymer domains for self-adhesive ability. This hybrid structure provides a low sheet resistance (<10 Ω sq^–1^) and maintains electrical performance under high strains exceeding 180% and has been applied to a 4 μm ultrathin EMG electrode array capable of detecting hand gestures.

While extensive hybrid strategies aim for electrical-mechanical synergy, Ma et al. developed a moisture- and air-permeable liquid-metal fiber mat (LMFM) by printing EGaIn onto an electrospun poly­(styrene-*block*-butadiene-*block*-styrene) (SBS) fiber mat, followed by mechanical activation via cyclic prestretching (Figure 7e).^87^ The prestretching process induces the formation of a laterally mesh-like and vertically wrinkled structure, suspended within the porous fiber network. This design facilitated ultrahigh stretchability (>1800%) and exceptional permeability to air and moisture. LEDs integrated into the LMFM remain fully functional under stretching and twisting, demonstrating the robust electrical reliability of the printed interconnects. Notably, the LMFM did not induce erythema after 1 week of skin attachment, indicating its potential for long-term wearability of sweat sensors.

To provide a clearer comparative perspective, Table 1 summarizes representative sensor architectures and their associated materials with respect to key parameters such as thickness, stretchability, durability, and different application domains. By organizing planar, porous, and composite platforms, the table highlights both material- and structure-dependent trade-offs and guides readers in identifying suitable design strategies for different wearable sensing applications.

**Table 1:** Comparison of Sensor Architectures and Materials with Respect to Mechanical Properties and Applications

structures	materials	thickness	stretchability	durability	application	refs
planar	graphene	2 nm	100%	20,000 cycles at 100% strain	pressure-sensing skin electronics	^65^
	sing-crystalline GaN	200 nm	10.3%	3000 cycles under bending loading	SAW Ion and UV sensors	^67^
	parylene/MXene	300 nm	30%	5000 cycles at 30% strain	ECG, EMG monitoring	^68^
porous	EGaIN/PU/ε-PL	80 μm	500%	15% resistance change after 1000 cycles at 500% strain	ECG, ICG monitoring	^69^
	cross-linked polyimide aerogel fiber	300 μm (diameter)	>20%	96.7% of mass retention ratio at 50 heating–cooling cycles	thermoregulating clothes	^70^
	AgNWs/PLGA/PVA	120 μm	>350%	50,000 cycles of 40 N loading at 3 Hz	physiological body monitoring	^71^
composite	graphene/PEDOT:PSS	100 nm	40%		ECG, sEMG	^83^
	laser-induced graphene/polyvinyl alcohol (PVA)–phytic acid (PA)–honey (PPH) hydrogel	1.0–1.5 μm	220%		ECG, heart rate, temperature, room humidity, cardiac patch	^86^
	PDMS/gold	1.3 μm	300%	1.7% resistance increase at 0% strain after 5000 cycles at 100% strain	ECG, implantable neural interface	^80^
	SEBS/gold nanoparticles	∼100 μm	600% (mechanical stretchability)	600 cycles under tensile, bending, and twisting loading	in vivo neuromodulation, EMG	^81^
			180% (electrical stretchability)			
	poly(styrene-*block*-butadiene-*block*-styrene) (SBS)/eutectic gallium–indium alloy (EGaIn)	320 μm	1800%	18% resistance increase after 100 cycles at 1000% strain	ECG	^87^

### 3.2 Wearable Strategies for Soft Sensors

To maintain the stable operation of sensors, diverse materials and structures are introduced. For real-world applications, more practical properties like breathability or long-term stability are considered. In this approach, the diverse wearable strategies like printing methods^20^ and smart adhesives^89^ have emerged as powerful methods for a user-friendly fabrication process and comfortable integration of sensors onto complex biological surfaces.

#### 3.2.1 Printed Electronics and Electronic Tattoos

Printed electronics have contributed significantly to the development of wearable technologies due to their low-cost fabrication, scalable processing, and substrate versatility (Figure 8).^90^ However, conventional devices based on rigid or semiflexible substrates show poor mechanical compatibility with skin, leading to signal degradation and detachment under motion or perspiration.^91,92^

**Figure 8:**
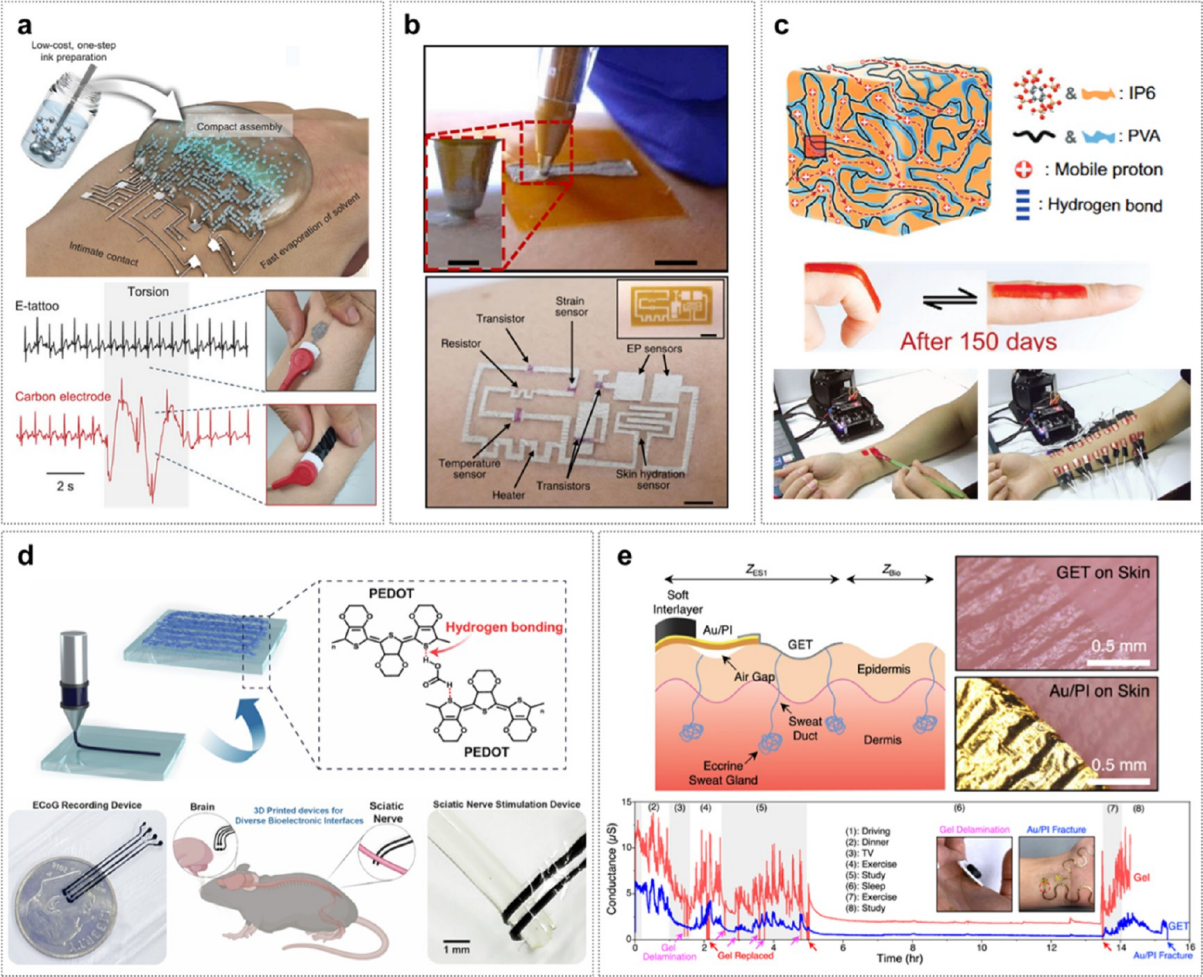
E-tattoo technologies for soft sensors. (a) Schematic illustration and photographs of direct assembly of the CMP on the skin for e-tattoos by the drawn-on-skin method (top), and comparison of CMP-based e-tattoos and carbon electrode for real-time ECG monitoring under mechanical deformation (bottom). Reproduced with permission from ref 93. Copyright 2022 Wiley-VCH. (b) DoS electronics drawing process with a modified ballpoint pen, and inks (top), and an example of an integrated system including various sensors and heaters drawn on human skin (bottom). Reproduced from ref 94. Available under a CC-BY 40 license. Copyright 2020 Ershad et al. (c) Schematic illustration of the materials of SF-supra-ICE (top), photographs of the mechanical properties under ambient air for 150 days (middle), and EMG-based human-machine interface system using SF-supra-ICE (bottom). Reproduced with permission from ref 95. Copyright 2023 Wiley-VCH. (d) Schematic illustration of PILC ink formulation (top), implantable bioelectronics PILC devices for ECoG recording, and nerve electrical simulation (bottom). Reproduced from ref 96. Available under a CC-BY 40 license. Copyright 2024 Oh et al. (e) Schematic illustration and image of GET electrode-skin interface (top), and long-term EDA sensing compared with commercial gel electrode (bottom). Reproduced from ref 20. Available under a CC-BY 40 license. Copyright 2022 Jang et al.

To overcome the limitations of conventional printed electronics approaches, e-tattoos have been developed. Their submicrometer thickness, low mechanical stiffness, and excellent breathability enable stable and high-fidelity signal acquisition, even under continuous skin deformation. These unique structural and mechanical properties allow e-tattoos to be applied across a wide range of applications, including biosignal monitoring.^20^ Graphene e-tattoos (GETs) for unobstructive ambulatory electrodermal activity sensing on the palm enabled by heterogeneous serpentine ribbons, temperature^97^ and strain sensing,^93^ and electrical stimulation.^96^ However, their extremely thin and delicate nature limits reusability, and current fabrication methods, such as transfer printing, remain complex and challenging to scale up.^98^

To create e-tattoo sensors, diverse fabrication methods have been proposed, including cut-and-paste,^99^ printing,^100^ and transfer printing^101^ techniques. Among these approaches, the drawn-on-skin (DoS) platforms represent a significant advancement, enabling in situ fabrication of e-tattoos directly on the skin without the need for substrate release or transfer process. With intrinsic simplicity, broad material compatibility, and excellent conformability to microscale skin features, the DoS method offers a user-friendly, rapid, and cost-effective route for e-tattoo manufacturing. As shown in Figure 8a, Lee et al. developed a substrate-free e-tattoo by assembling a composite material comprising platinum-decorated carbon nanotubes (CNT@Pt) and gallium-based liquid metal particles, referred to as CMP.^93^

This intrinsically conductive ink exhibits excellent wettability and rapid solvent evaporation, enabling uniform and conformal coating directly on skin surfaces within 10 s. The presence of CNT@Pt not only enhances electrical conductivity without postprocessing but also provides mechanical reinforcement, allowing the structure to endure dynamic deformation without delamination or signal degradation. Furthermore, the nanoporous structure allows for gas permeability, minimizing skin irritation during prolonged wear. These features make CMP highly favorable for soft sensing applications that require high-fidelity signal acquisition under motion and sweat-prone conditions.

Similarly, Ershad et al. demonstrated a pen-based DoS method that enables on-demand fabrication of multifunctional electronic circuits directly on human skin without the need for prefabricated substrates or printing equipment (Figure 8b).^94^ Using stencil-guided ballpoint pens and functional inks, including conductive (Ag-PEDOT:PSS), semiconducting (P3HT nanofibrils), and dielectric (ion gel) formulations, they sequentially drew ultrathin integrated devices on the skin. This approach simplifies fabrication while ensuring conformal integration under real-life conditions, including movement, sweating, and surface irregularities. Moreover, the authors demonstrated transistors, strain sensors, temperature sensors, heaters, hydration, and electrophysiological sensors by using the unified DoS method with multiple functional inks. The resulting sensors conform closely to skin microtopography, resist delamination under motion or perspiration, and maintain stable electrical performance. These characteristics contribute to low interfacial impedance and reliable signal acquisition, making the approach highly suitable for sensing applications that demand environmental robustness.

In contrast to previous approaches, Niu et al. proposed an ion-conductive electronic tattoo using a solvent-free supramolecular elastomer (SF-supra-ICE) composed of PVA and inositol hexakisphosphate (IP6) (Figure 8c).^95^ The material mimics the ion transport mechanism of human skin, enabling soft bioelectronics that operate in harmony with ionic signaling. The SF-supra-ICE demonstrates high ionic conductivity (>4.5 × 10^–2^ S·m^–1^), skin-like elasticity (Young’s modulus ∼0.3 MPa), strain stiffening, stretchability up to 573%, and self-adhesive behavior, all of which are crucial for stable signal acquisition under dynamic motion. These features ensure low interfacial impedance, minimal motion artifacts, and long-term biocompatibility, making it a platform for soft sensing in real-life electrophysiological monitoring and high-density EMG arrays.

Moving beyond 2D DoS techniques, Oh et al. developed a 3D e-tattoo platform using a highly conductive and biocompatible PEDOT:PSS-ionic liquid colloidal (PILC) ink (Figure 8d).^96^ This material’s composition, enabled by ionic liquid-induced phase separation and hydrogen bonding, exhibits excellent rheological properties for omnidirectional 3D printing, with high conductivity (∼286 S cm^–1^), mechanical robustness, and resolution down to 50 μm, while eliminating the need for post-treatment. Notably, this platform supports high-fidelity EMG and ECoG recordings on skin, as well as implantable spike arrays for neural interfaces and sciatic nerve stimulation under ultralow voltage for multifunctional sensing systems.

While the DoS method offers rapid and user-friendly prototyping, it is inherently limited in film thickness and structural resolution. In contrast, transfer printing, where microscale device components are fabricated on a donor substrate and then transferred onto the skin, enables the formation of ultrathin films with exceptional conformability and electrical performance with minimized skin-electrode impedance.^101–103^

In this context, Jang et al. developed a GET via transfer printing with a total thickness of 300 nm, achieving van der Waals-mediated adhesion to the skin (Figure 8e).^20^ Compared to traditional Au-based epidermal electrodes, graphene offers several advantages, including higher flexibility and biocompatibility, as well as reduced interfacial capacitance. Moreover, the use of serpentine geometries and heterogeneous layered design (HSPR) further improves strain tolerance and mechanical resilience. As a result, GETs achieve optical and mechanical imperceptibility, preserve skin breathability, and enable stable electrodermal activity (EDA) monitoring.

Each fabrication approach for printed e-tattoos presents distinct advantages and limitations in scalability, resolution, conformability, and long-term stability. Transfer-based methods enable ultrathin, high-performance devices with excellent skin adhesion but are costly and technically demanding, whereas direct writing strategies, such as drawn-on-skin, are rapid and material-versatile yet constrained by thickness control and structural resolution. These contrasts suggest that fabrication techniques should be carefully chosen based on the specific application rather than seeking a one-size-fits-all solution. Future advances in materials engineering and processing are expected to bridge these trade-offs, paving the way for robust, multifunctional, and user-friendly e-tattoo platforms.

#### 3.2.2 Smart Sensory Adhesives

In wearable sensor technologies, extensive efforts have focused on achieving stable biosignal monitoring, minimizing the signal-to-noise ratio. However, robust integration of electronic devices with biological surfaces remains challenging due to the complex and dynamic nature of biointerfaces. Skin possess inherently soft moduli, curved geometries, microscale wrinkles, and hairs, and is often moist, all of which hinder conformal and intimate contact, which is critical for reliable signal transduction and long-term functionality. To address these challenges, recent research has focused on improving mechanical compliance and interfacial adhesion by the incorporation of ultrathin designs. Owing to their high flexibility, ultrathin sensors can closely conform to deformable surfaces and increase the effective contact area and strengthen intermolecular forces at the sensor–tissue interface.

However, van der Waals interactions alone are often insufficient to maintain robust and durable adhesion under dynamic motions of biointerfaces. To overcome these limitations, smart adhesives have been developed that offer stimuli-responsive and reversible adhesion. The smart adhesive systems dynamically modulate interfacial bonding strength in response to stimuli such as temperature, humidity, or light.^104^ These dynamic responses are crucial not only for minimizing skin irritation and facilitating device reuse,^105–107^ but also for achieving conformal contact with complex biological tissues.^16,108^ Moreover, such adaptive behavior facilitates the transfer and stable integration of ultrathin wearable sensors onto various tissue surfaces.^89,109,110^ Realizing effective biointegration of ultrathin smart adhesives requires advances in tunability, multistimuli responsiveness,^111^ and wet adhesion performance.

Reversible adhesives that allow residue-free and nondamaging removal from delicate skin are essential for the long-term usability and safety of wearable systems. For example, Zhang et al. developed a photoresponsive hydrogel adhesive composed of cellulose nanofibers (CNFs), poly­(acrylic acid) (PAA) chains, Fe^3+^ ions, and dopamine (DA)-based adhesive functional groups (Figure 9a).^105^ Carboxyl groups in PAA and oxidized CNF form strong coordination complexes with Fe^3+^ ions, enhancing mechanical robustness. Catechol groups in DA promote adhesion through hydrogen bonding, π–π interactions, and covalent bonding with amine, hydroxyl, and carboxyl moieties on biological surfaces. Upon UV exposure, Fe^3+^ is photoreduced to Fe^2+^ via a photo-Fenton-like reaction, weakening the coordination network and inducing a supramolecular phase transition. This redox-responsive disassembly reduces tensile adhesion from 74.8 to 13.10 kPa, enabling gentle detachment from the skin. Integration with a TENG demonstrates its utility as a detachable, conformal, self-powered bioelectronic interface for real-time physiological monitoring.

**Figure 9:**
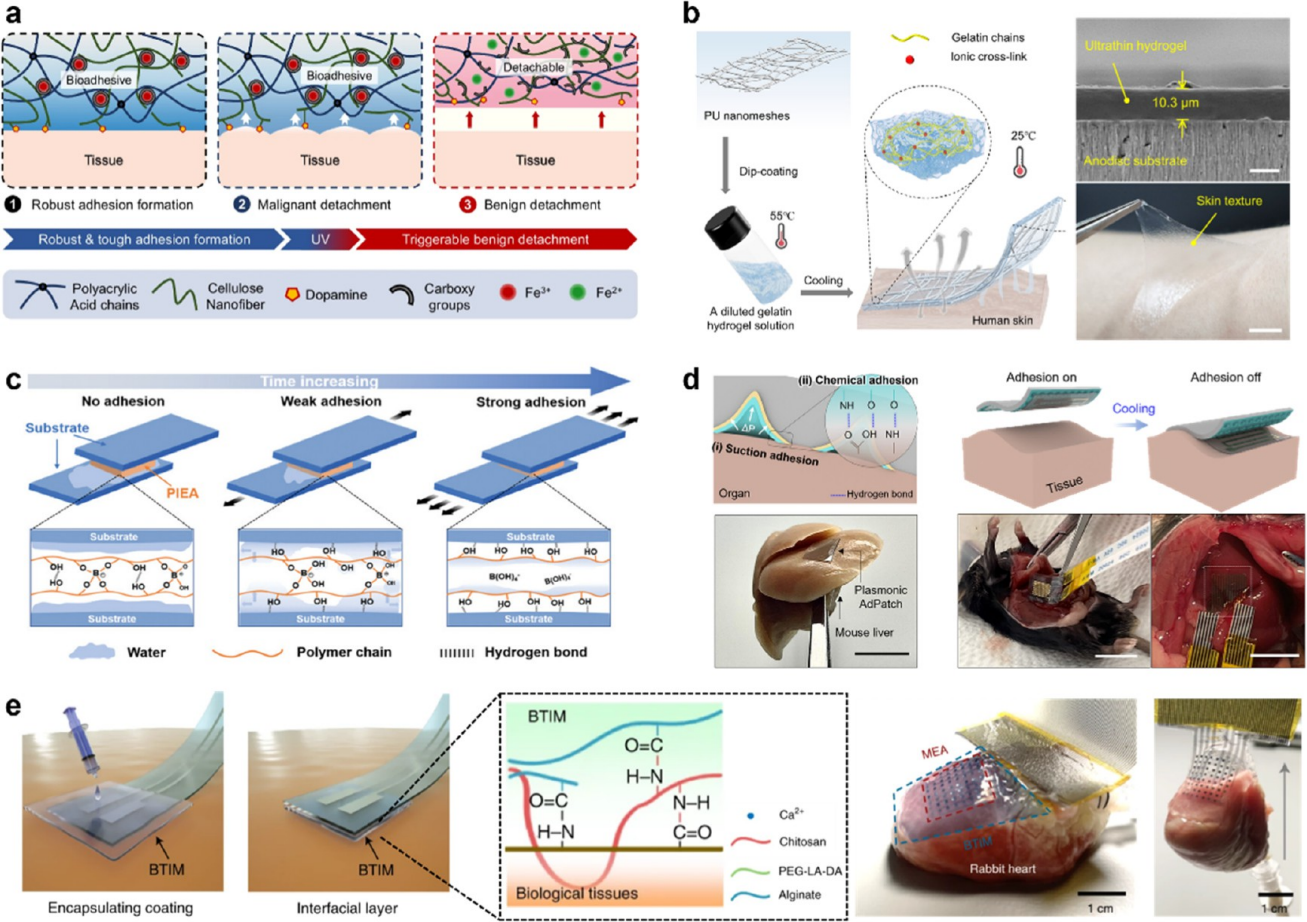
Smart sensory adhesives. (a) Photoresponsive adhesion mechanism on tissue, showing reversible bonding via Fe^3+^/Fe^2+^ redox. Reproduced from ref 105. Available under a CC-BY 40 license. Copyright 2024 Zhang et al. (b) Thermal-responsive adhesive based on a PU nano mesh embedded in gelatin (left). Cross-sectional SEM image of the ultrathin hydrogel and photograph showing peel-off from human skin (right). Reproduced from ref 16. Available under a CC-BY 40 license. Copyright 2024 Zhang et al. (c) Water-enhanced adhesion mechanism under aqueous conditions, inducing strong hydrogen bonding between polymer and substrate. Reproduced with permission from ref 112. Copyright 2024 Wiley-VCH. (d) Suction and chemical adhesion mechanisms on the organ. Photographs of smart adhesive applied to the mouse liver (left). Schematic and photograph of the ultrathin sensor transfer using the smart adhesive (right). Reproduced from ref 89. Copyright 2024 American Chemical Society. (e) Photocurable adhesive interfaces between electronic devices and biological tissues (left). Photograph showing stable integration of an ultrathin sensor onto a rabbit heart via photocurable adhesives (right). Reproduced with permission from ref 113. Copyright 2021 Springer Nature.

It is important to note that achieving conformal contact on irregular skin topography remains challenging due to microscale hairs and air gaps. To establish a conformal interface between electronics and biological components, phase-transition biogels as adaptive adhesives have been explored.^107,114,115^ Modulus engineering enables them to adapt to microscale interfacial gaps on biological interfaces, allowing conformal integration with skin topography.

In related research, Zhang et al. developed an ultrathin biogel reinforced with a PU nanomesh embedded in a gelatin matrix (Figure 9b).^16^ Gelatin contains abundant amino acids and charged groups that enable strong interfacial bonding through hydrogen bonding, electrostatic interactions, and complexation bonding, achieving a skin adhesion energy of 176.8 μJ cm^–2^. With a reduced thickness of ∼10 μm and a modulus comparable to that of human skin, the biogel provides excellent conformability to irregular surfaces. Furthermore, the gelatin network exhibits a reversible sol–gel transition, enabling easy detachment upon mild heating above 55 °C. This property is critical for applications in hairy regions such as the scalp.

Moreover, stable adhesion in wet environments remains a major challenge due to the persistent interfacial water layer, which hinders direct contact and bonding. To overcome these issues, water-tolerant adhesives have emerged as a promising strategy to facilitate spontaneous and durable adhesion under wet conditions.^116,117^ In fact, Li et al. reported a water-responsive smart adhesive synthesized by copolymerizing a hydrophobic ionic liquid (1-hydroxypropyl-3-vinylimidazolium bis­(trifluoromethanesulfonyl)­imide ([HPVIm]­[TFSI])), 2-methoxyethyl acrylate, and boric acid (Figure 9c).^112^ Initially nonadhesive in air, the surface undergoes hydrolysis of dynamic borate ester bonds upon immersion in water. The resulting hydroxyl groups migrate to the interface and form strong hydrogen bonds with the substrate, thereby achieving an underwater adhesion strength as high as 314 kPa, thus facilitating the water-responsive smart adhesives offer valuable potential for applications in underwater sensing and biomedical interfaces.

Extending beyond the skin, the integration of bioelectronic devices with internal biological tissues offers capabilities for implantable systems.^93^ Although ultrathin sensors offer enhanced mechanical compliance and conformal contact with soft biological interfaces, their fragile structure poses significant challenges during transfer. For example, Kim et al. developed an octopus-inspired smart adhesive composed of temperature-responsive poly­(*N*-isopropylacrylamide) (PNIPAM) hydrogel and photothermal gold nanostars on a hole-patterned PDMS substrate (Figure 9d).^89^ Upon exposure to near-IR (NIR) light or elevated temperature, the PNIPAM hydrogel contracts, thereby enhancing suction adhesion through localized negative pressure, thus causing strong adhesion of 10 kPa on a mouse liver. Moreover, utilizing its light-responsive tunable adhesion properties, the smart adhesive enabled the successful transfer of 3 μm-thick sensors onto the liver by tuning adhesion. This controllable adhesion allows for the facile relocation and precise placement of ultrathin sensors without damage.

The smart adhesive system not only facilitates the easy transfer of ultrathin sensors but also enables robust integration with biological interfaces. Indeed, Yang et al. reported a photocurable adhesive that enables robust integration of bioelectronic devices with soft tissues (Figure 9e).^113^ To achieve conformal bonding to organ surfaces, the interpenetrating network composed of polyethylene glycol-lactide diacrylate and sodium alginate was developed for photocuring. Chitosan was incorporated as a tissue primer to promote strong interfacial adhesion, in which the primary amine groups form covalent bonds with carboxylic acid groups on both the tissue surface and the alginate network. The liquid precursor conforms to complex tissue surfaces and undergoes light-induced cross-linking into a ∼500 μm-thick elastic adhesive, forming a strong adhesion on skin and on epicardial tissue. The adhesive enabled stable integration of ultrathin multielectrode arrays onto the rabbit heart, allowing reliable ECG signal monitoring for up to 8 h.

Biological interfaces undergo continuous deformations in different modes such a stretching, compression, and bending, which necessitate strong and reliable sensor–tissue integration for long-term operation. While robust adhesion ensures stable signal acquisition, it often raises concerns regarding skin damage or residues during detachment. Therefore, the development of reversible adhesive strategies is crucial for wearable systems such as they can attach to the skin during operation while allowing gentle detachment upon external stimuli. The intensity of these external triggers must be carefully engineered to avoid harsh conditions that could damage delicate biological tissues.

## 4 Sensor Materials and Structures Applications

### 4.1 Tactile Sensors

To date, numerous studies have explored tactile sensors using a wide range of materials, structural designs, and applications.^118–123^ Compact, miniaturized, and portable systems offer a promising direction for real-world implementation, including virtual and augmented reality usage.

#### 4.1.1 Tactile Sensor Using Piezoresistive and Capacitive Mechanisms

Piezoresistive and capacitive sensing mechanisms are widely used due to their simplicity and signal accuracy. Piezoresistive sensors are relatively easy to fabricate, typically by placing a conductive layer between two dielectric layers. Resistance is then measured and correlated to the applied pressure, providing a tactile output. It is important to note that since piezoresistive sensors are passive sensors, an external power supply is required to read out signals from the piezoresistive sensors.^124,125^

In a recent study, Kim et al.^79^ suggested ultrathin wearable electronic skin for three-dimensional tactile interfaces (Figure 10a). The design contains a multiwalled carbon nanotube (MWCNT) covered with elastomeric capping layers. The fabricated sensor has a thickness of 50 μm, which is skin-conformable and highly stretchable. By measuring the impedance of fabricated devices, three-dimensional tactile sensing can be achieved through the suggested device.

**Figure 10:**
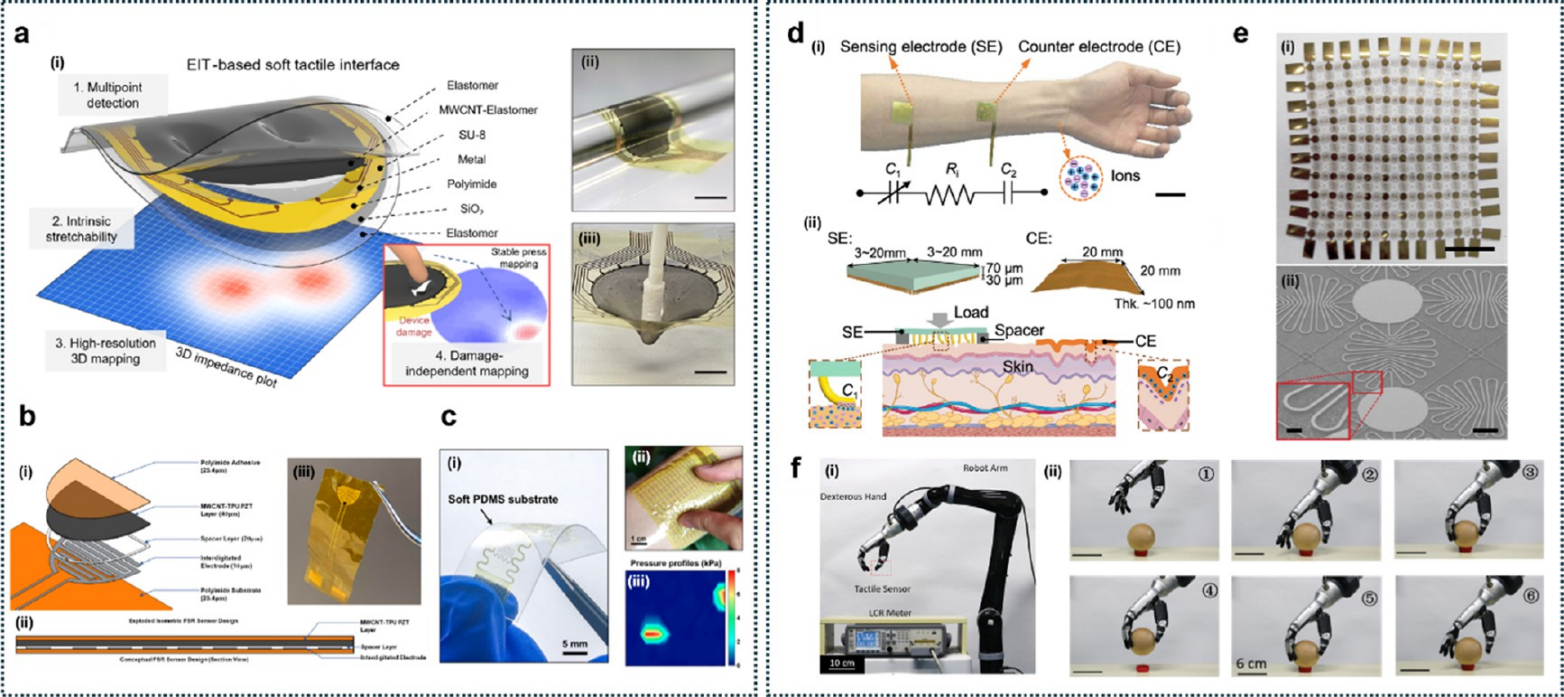
Tactile sensors use piezoresistive and capacitive sensing. (a) (i) Intrinsically stretchable, interconnect-free soft e-skin for high-resolution 3D tactile mapping via electrical impedance tomography (EIT). (ii) Photograph of the soft tactile sensor bent over a rod. Scale bar, 5 mm. (iii) Photograph of the device pressed by a swab, demonstrating the intrinsically stretchable property of the sensing area. Scale bar, 5 mm. Reproduced from ref 79. Available under a CC-BY 40 license. Copyright 2024 Kim et al. (b) Schematic illustration of multilayered resistive tactile sensor design at (i) top view and (ii) side view. (iii) Picture of fabricated tactile sensor. Reproduced from ref 126. Copyright 2023 American Chemical Society. (c) (i) Optical image of the flexible e-skin prototype. (ii) Optical images of the e-skin mounted onto the forearm pressed by two fingers. (iii) The pressure and profiles given by the e-skin. Reproduced from ref 127. Available under a CC-BY 40 license. Copyright 2021 Cai et al. (d) (i) Photograph of the SEMS with a SE and a CE laminated on an arm, and a simplified equivalent circuit of the SEMS. Scale bar: 5 cm. (ii) Exploded view and schematic illustration of the SEMS. The insets of the schematic show the skin-electrode interface upon loading. Reproduced from ref 128. Available under a CC-BY 40 license. Copyright 2021 Zhu et al. (e) (i) Optical image of the fabricated polyimide network (10 × 10 array, scale bar: 5 mm). (ii) Tilted SEM image of the polyimide network (scale bar: 500 μm); the inset is a higher resolution SEM image of a meandering interconnect (scale bar: 50 μm). Reproduced from ref 129. Available under a CC-BY 40 license. Copyright 2018 Hua et al. (f) Applications of the tilted microhair array-based tactile sensors integrated with a robot hand for grasping experiments. (i) Photograph of the experimental system. (ii) Stepwise process of the robot hand during grasping a wooden ball: approaching, touching, grasping, lifting, lowering, and releasing. Reproduced from ref 130. Available under a CC-BY 40 license. Copyright 2024 Yu et al.

To this end, Aubeeluck et al.^126^ developed a screen printing-based piezoresistive tactile sensor with the MWCNT as a conductive layer. The design of the suggested tactile sensor includes an interdigitated electrode with an MWCNT-thermoplastic polyurethane (TPU) composite covered with a polyimide layer (Figure 10b). Through the screen-printing method, the final thickness of the tactile sensor is 110 μm, and the sensor can have an advantage in its scalability to the connected arrays for the fabricated sensors. Figure 10c shows a piezoresistive sensor comprising Cr/Au pixel electrodes sandwiched between two PI layers atop a PDMS substrate.^127^ This architecture allows for high-resolution pressure sensing across multiple pixels and also enables temperature sensing by detecting the thermal expansion of the soft substrate.

Capacitive ultrathin tactile sensors operate by detecting changes in capacitance, typically driven by pressure-induced volumetric changes in the intervening dielectric material. While fabricating these sensors in ultrathin formats can be more challenging, their primary sensing mechanism hinges on how the dielectric layer compresses or deforms under pressure. A key consideration for thin-film structures is that the inherent volumetric change may be less dynamic, potentially limiting their sensing range and linearity. However, their ability to achieve high sensitivity has been demonstrated, making them well-suited for sensitive applications.^131,132^

As an illustration, Figure 10d depicts a skin-conformable capacitive sensor where a sensing electrode (SE) interacts with a counter electrode (CE).^128^ While the entire system is not fully skin-conformable, the SE detects tactile stimuli via the deformation of gold-coated micropillars. Introducing microstructures has been applied to various applications,^133^ allowing pressure imaging across the glove surface. Hua et al.^129^ suggested another multilayered sensor for multifunctional sensing. By adding up layered structures of different arrays, multifunctional detection can be achieved. They fabricated a stretchable and conformable matrix network structure composed of 100 sensory nodes connected by meandering wires. The unique meandering wire structure makes the device stretchable, which can be operated at a conformable contact to the human skin (Figure 10e). Tactile sensing is achieved by measuring the capacitance as the distance between two Ag electrodes reduces. With a skin-inspired structure, conformable devices can also be used as human-machine interfaces to collect signals and assist the robotic movement.

Finally, Yu et al.^130^ introduced a capacitive tactile sensor having a microhair array in between two electrodes to assist a robotic hand to grasp different objects (Figure 10f). Conformable design makes it easier to apply on various robotic surfaces, which can be crucial for future applications such as automated assembly, where precise pressure measuring and feedback are crucial.

#### 4.1.2 Tactile Sensor Using Piezoelectric, Triboelectric, and Piezoionic Mechanisms

While piezoresistive and capacitive systems benefit from straightforward fabrication and material choices, self-powered tactile sensors offer distinct advantages by eliminating the need for external power sources, simplifying device integration, and extending operational lifespan. Common mechanisms exploited to date include piezoelectric and triboelectric effects, which generate electrical signals directly from mechanical deformation or contact-separation. More recently, piezoionic systems are also gaining attention for their minimalistic architecture, relying solely on optimized polymer matrices and ion movement to achieve highly sensitive tactile sensing.^134,135^

Piezoelectric tactile sensors are realized using materials that generate an electrical charge when mechanically stressed (Figure 11). These materials can be broadly categorized into inorganic piezoelectric ceramics like aluminum nitride (AlN), zinc oxide (ZnO), lead zirconate titanate (PZT), and barium titanate (BaTiO_3_), or piezoelectric polymers such as polyvinylidene fluoride and its copolymers, doped polyimide, and poly-l-lactic acid.^136–140^ When fabricating ultrathin, skin-conformal tactile sensors with inorganic materials, common approaches involve depositing nanometer-thick films, which are then carefully lifted off their substrates. Conversely, if piezoelectric polymers are chosen, various established thin-film fabrication methods, including spin coating, electrospinning, and dip coating, can be employed to achieve the desired ultrathin form factor for highly flexible and adaptable sensors, critical for applications like electronic skin for health monitoring.^141^

**Figure 11:**
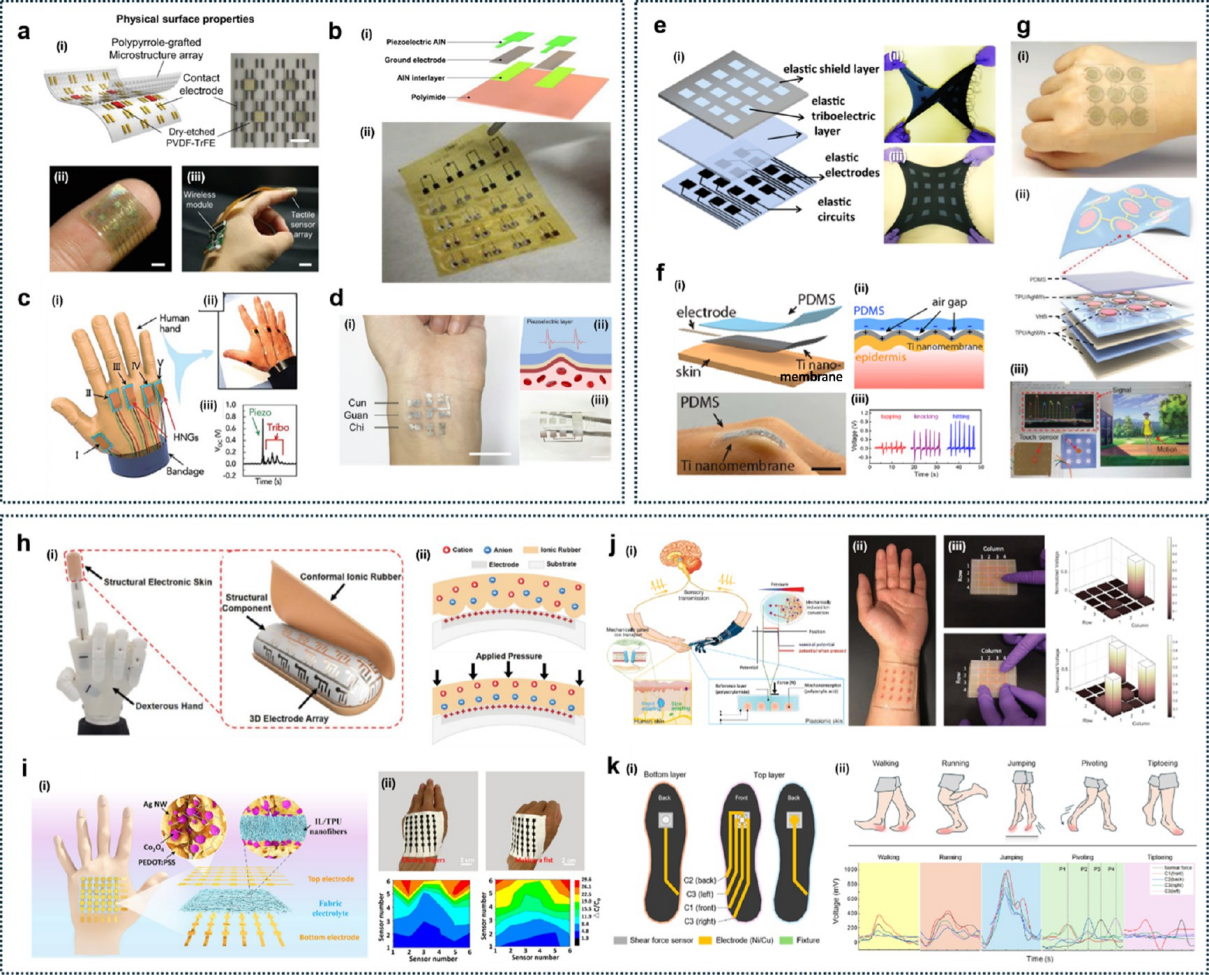
Tactile sensors for piezoelectric, triboelectric, and piezoionic sensing. (a) Ultraflexible bimodal (UFB) tactile sensor for telehaptic system. (i) 3D schematic of UFB tactile sensor array and optical microscopy image of the UFB tactile sensor array, scale bar is 2 mm. (ii) Image of the UFB tactile sensor laminated on the fingertip, scale bar is 2 mm. (iii) UFB tactile sensor with a wireless system attached to the hand for tactile acquisition, scale bar is 2 cm. Reproduced from ref 142. Available under a CC-BY 40 license. Copyright 2022 Jin et al. (b) (i) Fabrication design of a piezoelectric stack of AlN-IL/Mo/AlN grown on PI 2555. (ii) A series of different sensors varying their diameter. Reproduced from ref 143. Available under a CC-BY 40 license. Copyright 2023 Carluccio et al. (c) (i) Representation and (ii) photo of a human hand with five wearable hybrid sensors attached to its back. (iii) Voltage signal for the full extension of the index finger. Reproduced with permission from ref 144. Copyright 2021 Wiley-VCH. (d) (i) Photograph of the device attached to the skin for monitoring the arterial pulse at Cun, Guan, and Chi positions (scale bar is 20 mm). (ii) Schematic illustration of testing arterial pulse using piezoelectric films. (iii) Photograph of the ultrathin P­(VDF–TrFE) piezoelectric film. Reproduced from ref 145. Copyright 2023 American Chemical Society. (e) (i) The exploded view of the UTE-skin architecture involving elastic circuits, elastic electrodes, elastic triboelectric layer, and elastic shielding layer. (ii) Photographs of a fabricated triboelectric sensor array-based e-skin under twisting and stretching, demonstrating its mechanical robustness. Reproduced from ref 147. Available under a CC-BY 40 license. Copyright 2024 Shao et al. (f) (i) Schematic illustration of the on-skin TENG with the Ti nanomembrane and photo of the on-skin TENG. Scale bar, 1 cm. (ii) Schematic illustration of the cross-section of the on-skin TENG. (iii) Output voltage of the on-skin TENG when the hand touches the rigid surface with different forces. Reproduced from ref 148. Copyright 2023 American Chemical Society. (g) (i) Picture showing that the skin-inspired TENG (SI-TENG) is attached to the hand. (ii) Scheme diagram of the 3 × 3 SI-TENG array and the detailed structure of the 3 × 3 SI-TENG array. (iii) Demonstration showing that the SI-TENG array controls game character movements. Reproduced with permission from ref 149. Copyright 2021 Wiley-VCH. (h) (i) The photo of the structural electronic skin (SES) integrated with a dexterous hand and the structure of the SES. (ii) The pressure-sensing mechanism of the SES. Reproduced from ref 150. Available under a CC-BY 40 license. Copyright 2023 Li et al. (i) (i) Schematic diagram of the integrated all-nanofiber tactile sensor. (ii) Photographs and corresponding spatial relative capacitance change distribution acquired when the hand is closing and is making a fist. Reproduced with permission from ref 151. Copyright 2022 Elsevier. (j) (i) Mechanoreceptors have anchors that attach the membrane to the extracellular matrix as well as a cytoskeleton that both stretch an ion channel, causing an influx of sodium ions upon deformation. Once the cell membrane potential increases, voltage-sensitive ion channels (not shown) open to allow reversal to the resting potential. The rapid- and slow-adapting mechanoreceptors differ, in part, in the mechanical structures of the cell. Piezoionic skin assembly has a built-in potential difference set by the difference in fixed charge concentration between poly­(acrylic acid) (polyAA; charged) and polyacrylamide (pAAM; neutral). Compression of the charged side creates a flux of solvent and protons that increases this potential difference. (ii) Photograph of a 16-element piezoionic mechanoreceptor array on a wrist, demonstrating conformability. (iii) Photograph and corresponding normalized voltage bar plot of the piezoionic mechanoreceptor array detecting a single touch (top). Photograph and corresponding normalized voltage bar plot of the piezoionic mechanoreceptor array detecting multiple touches (bottom). Reproduced with permission from ref 152. Copyright 2022 The American Association for the Advancement of Science. (k) (i) Schematics showing the electrode connection of the piezoionic porous shear force sensor inside the shoe insole. (ii) Sensor response to different foot motions (walking, running, jumping, pivoting, and tiptoeing) showing different peak intensities and signals at different connections (C1, C2, C3, and normal force). Reproduced from ref 157. Copyright 2025 American Chemical Society.

Figure 11a presents a pixelated piezoelectric telehaptic system integrating a tactile sensor interface and a piezoelectric actuator interface.^142^ The sensor uses P­(VDF–TrFE) with polypyrrole-grafted thin film arrays, connected with the actuator comprises a multilayer PZT array on a PI substrate. This integrated thin film system offers great promise for virtual reality and augmented reality applications through the skin-conformable design and wirelessly connected system.

Next, Carluccio et al. developed AlN-based piezoelectric tactile sensors with a thin polyimide substrate (Figure 11b).^143^ The authors deposited Mo as an electrode for collecting signals from the round-shaped AlN piezoelectric layer. The piezoelectric layers’ diameter can be controlled from 5 to 500 μm, which results in the impedance difference, showing the potential of piezoelectric materials as a tactile sensor over a broad pressure range.

Another example of using piezoelectric inorganic materials for skin-conformable devices is shown in Figure 11c, where, Mariello et al. developed a hybrid piezo/triboelectric wearable sensor by combining AlN and a porous PDMS/Ecoflex blend.^144^ The fabricated device can be made into an arrayed structure, which can be applicable to sensing hand gestures and monitoring gait cycles. For an example of the tactile sensor using piezoelectric polymers, Tian et al. developed an ultrathin epidermal P­(VDF–TrFE) film having an evaporated Ag electrode on the polymeric substrate (Figure 11d).^145^ The fabricated ultrathin film-based tactile sensor can detect arterial pulse, which can find potential applications as a sports monitoring system.

Similar to capacitive sensors, operating triboelectric devices in an ultrathin, skin-conformable format presents structural limitations, primarily because these sensors typically necessitate a spacer to facilitate contact-separation between oppositely charged layers. To circumvent this constraint, some researchers are exploring innovative single-electrode triboelectric devices. These designs cleverly leverage the inherent surface potential of human skin, which is positively charged, or the surface charge of other contacting materials as the second triboelectric layer. This approach enables the fabrication of truly ultrathin sensors, minimizing the need for complex internal spacing and enhancing skin conformability for applications like e-skin health monitoring.^147,153^

As an illustration, Figure 11e shows a 4 × 4 stretchable triboelectric sensor array made of Ecoflex and carbon black, demonstrating applicability in touchpads, smart gloves, and insoles.^147^ Park et al.^148^ demonstrated an ultrathin 2D titanium nanomembrane that can be conformably attached to human skin (Figure 11f). The attached nanomembrane can be combined into a triboelectric device by utilizing an air gap formed between a rough skin surface and a flat polymer surface. A fabricated sensor can sense the hand touches of different forces at the rigid surface. Next, Figure 11g shows the 3 × 3 triboelectric tactile sensor array targeted for use as a game controller.^149^ The sensor is composed of a PDMS electrification layer, TPU/AgNWs stretchable electrode layer, VHB insulation layer, and to add compatibility for a real application, TPU/AgNWs shielding layer, and VHB/TPU substrate layer are added. Once in contact with other materials, the modified PDMS film tends to gain negative triboelectric charges and promote touch sensitivity due to very low, 100 μm, thickness.

It is important to note that unlike traditional piezoelectric sensors that rely on charge generation from mechanical stress or friction, piezoionic sensors achieve tactile sensing through the dynamic redistribution of ions within a polymer electrolyte matrix. This unique operating principle allows for the creation of exceptionally flexible, conformable, and miniaturized devices, often with simpler fabrication pathways than their counterparts. Their ability to translate subtle mechanical stimuli into measurable ionic currents makes them ideal candidates for next-generation electronic skin and highly sensitive wearable technologies.^154,155^

As different examples, Figure 11h highlights a piezoionic tactile sensor using a conformal ionic rubber matrix.^150^ Applied pressure dissociates the ionic liquid into oppositely charged ions, forming an electric double layer measurable as a capacitance or potential difference. The sensor, featuring a 3D electrode array, detects pressure distribution with high sensitivity, capable of sensing lightweight materials like cotton, structural features as fine as human hair, and variably shaped objects. In a recent study, Wang et al.^151^ developed a piezoionic tactile sensor with an integrated all-nanofiber network structure (Figure 11i). The upper and bottom electrodes are composed of highly conductive (≈10^3^ S/cm) and flexible PEDOT:PSS, further enhanced with AgNWs. Co_3_O_4_ microspheres are added to improve sensitivity via their pseudocapacitance and microstructure. The sensor is fabricated through a simple two-step process: coelectrospinning of ionic liquid/TPU nanofibers and ink-patterning of electrodes on both sides. For the final application of the fabricated sensors, A 6 × 6 flexible sensor array was fabricated via ink-patterning for spatial pressure mapping, maintaining flexibility even at a large size (45 cm × 50 cm). It accurately captured 2D pressure distributions, enabling effective gesture recognition.

Next, Dobashi et al.^152^ demonstrated enhanced piezoionic mechanoreceptors based on hydrogels (Figure 11j). Fabricated piezoionic sensors mimic biological systems by using ionic currents to achieve high charge density and low-voltage operation, relying on pressure-driven ion flux. Unlike mechanoreceptors, signals are generated through selective ion transport or streaming within the polymer matrix.^156^ The authors demonstrated a 4 × 4 piezoionic sensor array, composed of poly­(AA-*co*-AAm) hemispheres surrounded by potassium chloride-swollen polyacrylamide (pAAm), which enables single- and multitouch detection with minimal crosstalk and ∼1 s transient response. Gentle touches (∼100 gf) produce ∼−10 mV signals superimposed on a −50 mV Donnan potential, with response tunable via film thickness, polymer content, or current-based readout.

In fields like robotics and gait analysis, it is crucial to measure both normal pressure and shear force simultaneously to accurately detect multidirectional mechanical deformation. Recently, Kang et al. sought to address this challenge by developing a self-powered piezo-ionic sensor with a unique two-layer structure, featuring an interlocked reversed trapezoidal structure and a macrodome structure.^157^ Based on porous TPU and an ionic liquid, this sensor converts externally applied shear force into localized compression, enabling the simultaneous detection of normal and tangential forces with high sensitivity over a wide dynamic range. The research team demonstrated its practical application by integrating the sensor into a shoe insole (Figure 11k). By embedding the sensor in the forefoot region of the insole and placing electrodes corresponding to forward, backward, and side directions (C1, C2, C3), they successfully measured directional changes in shear force according to the subtle movements of the foot. This has opened up new possibilities for precisely analyzing complex and dynamic foot movements such as walking, running, pivoting, and tiptoeing.

Overall, these skin-conformable tactile sensors hold great promise for next-generation electronic skin technologies, owing to their structurally simple design, ease of miniaturization, and excellent adaptability. Their flexible and scalable architecture makes them highly suitable for diverse applications, including humanoid robotics, human–computer interaction, and wearable biosensing platforms that require intimate and responsive skin-like interfaces directly on the body.

### 4.2 Self-Powered Sensors

For ultrathin soft wearable devices to be widely adopted in daily life as portable and body-attached devices, they require high flexibility and unobtrusive comfort that is not affected by motion and time. To meet these demands, ultrathin devices integrated with energy harvesting technologies are emerging as an advanced solution. These systems simplify overall architecture and enhance autonomy by eliminating reliance on external power sources or frequent battery replacement. Owing to their extremely thin and flexible form, such devices can conform intimately to a complex and dynamic body, much like a second skin, while providing imperceptible comfort.

Simultaneously, these sensors are engineered to maintain long-time durability against mechanical deformations from daily activities, while efficiently harvesting energy from body motion, thereby functioning as independent power sources for embedded electronics. To realize such self-powered ultrathin wearable sensors, active research is underway on innovative materials and structural designs to integrate various energy harvesting devices, including piezoelectric,^158^ triboelectric,^159^ and thermoelectric nanogenerators.^160^

Among self-powered materials, piezoelectric thin films have garnered significant attention for their ability to directly convert ambient mechanical energy, such as human motion, into reliable electrical signals. A key focus of current research is enhancing their mechanical compliance, particularly flexibility and stretchability, to enable seamless integration with the soft, dynamic nature of the human body. Furthermore, innovative micro- and nanoscale structural designs are being actively investigated to optimize the electromechanical conversion efficiency and overall durability.^161^

These research efforts are not only demonstrating potential efficient energy converters but also inherently suitable for intimate, long-term contact with the human body. Recently, Li et al. demonstrated a highly stretchable and biocompatible piezoelectric film, self-assembled from dl-alanine amino acid, which forms a unique truss-like microfiber network (Figure 12a).^162^ This structure allows the film to endure significant tensile strains (up to 40%) while maintaining its piezoelectric properties. A stretchable nanogenerator was fabricated by integrating this dl-alanine network with percolated Ag nanowire electrodes on a PDMS substrate. When stretched by 20% and subjected to oscillations, it consistently produced a peak-to-peak voltage of ∼90 mV. When applied to a human knuckle, it generated ∼60 mV during bending, and in vivo tests on swine muscle showed voltage outputs of 20 mV in response to leg movements, highlighting its potential for tissue-compatible biomedical devices.

**Figure 12:**
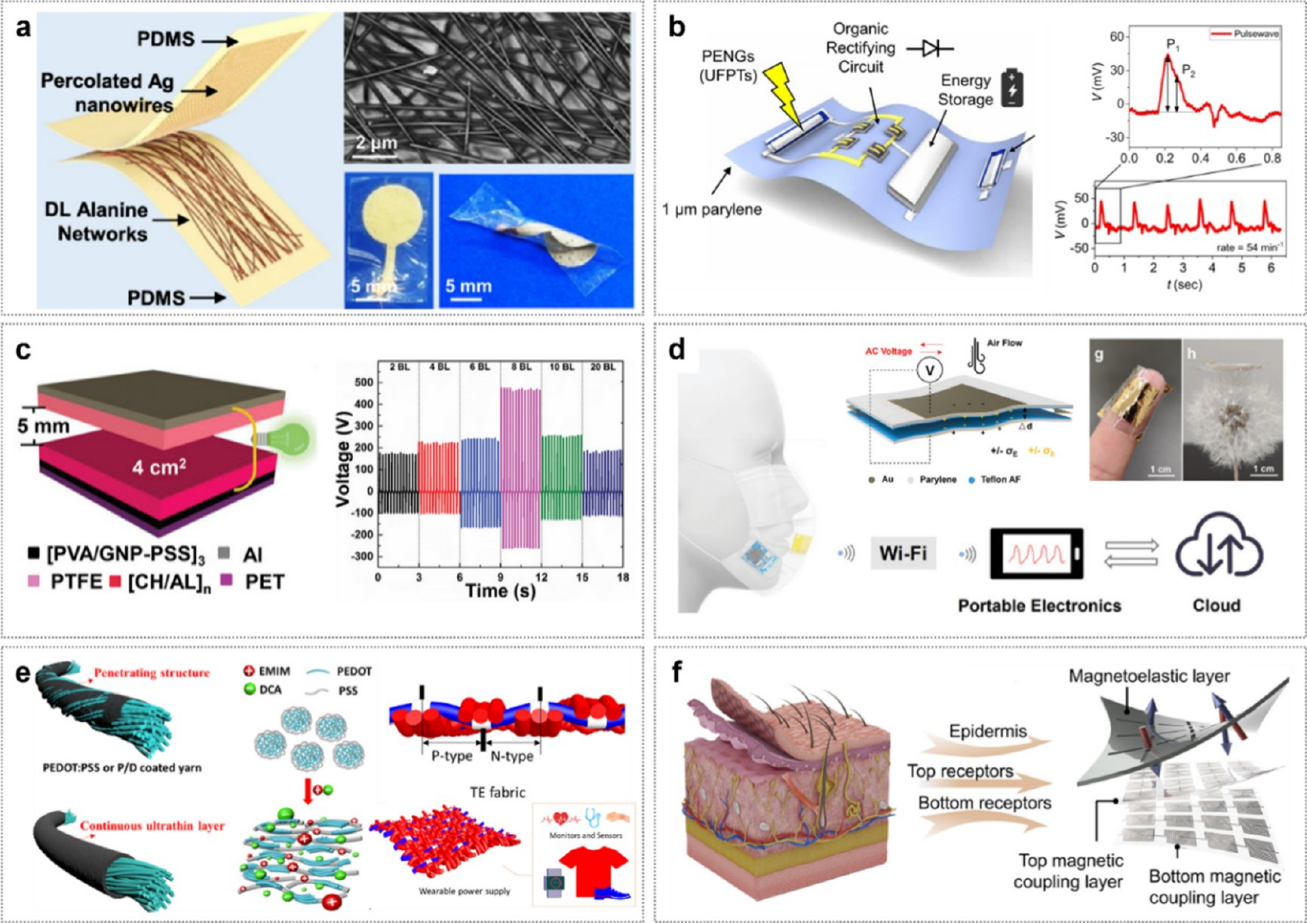
Ultrathin energy harvester. (a) Self-assembled dl-alanine biocrystal network-based piezoelectric nanogenerator. Reproduced from ref 162. Available under a CC-BY 40 license. Copyright 2023 Li et al. (b) Ferroelectric polymer transducers integrated on a thin parylene substrate for harvesting biomechanical energy. Reproduced from ref 163. Available under a CC-BY 40 license. Copyright 2023 Petritz et al. (c) Thickness-optimized TENG with layer-by-layer self-assembled ultrathin chitosan-alginate composite. Reproduced with permission from ref 164. Copyright 2023 Wiley-VCH. (d) Wireless smart face mask with ultrathin parylene-Teflon AF triboelectric nanogenerator. Reproduced with permission from ref 165 Copyright 2022 Wiley-VCH. (e) PEDOT:PSS/DMSO/Ionic liquid composite coated yarns for fabric-type wearable thermoelectric nanogenerator. Reproduced from ref 166 Copyright 2021 American Chemical Society. (f) Human somatosensory system-inspired magnetoelastic artificial skin for underwater haptic sensing. Reproduced from ref 167. Available under a CC-BY 40 license. Copyright 2024 Zhou et al.

While piezoelectric nanogenerators and rectifier diodes show great promise for energy harvesting and sensing, they often lack mechanical compliance and scalability for mass production.^166^ To address this issue, there is a growing demand for ultrathin, ultraflexible piezoelectric nanogenerators that can conform to the body, function as both power sources and sensors, and be produced at scale. Therefore, Petritz et al. introduced an ultraflexible energy harvesting sensor with a thickness of 2.5 μm, which integrates a piezoelectric nanogenerator, a diode-based rectifier, and a storage capacitor on an ultrathin (1 μm) flexible parylene substrate (Figure 12b).^163^

The core piezoelectric component consists of ultraflexible ferroelectric polymer transducers made from P­(VDF–TrFE) (70:30) with thermally evaporated Al electrodes, and organic full-wave rectifiers based on dinaphtho-thieno-thiophene (DNTT) organic thin film transistors. The P­(VDF–TrFE) transducers achieved a high remnant polarization (*P*_r_) of ∼67 mC m^–2^ and a transversal force sensitivity up to 1.3 nC N^–1^ on a silicone rubber carrier. The DNTT-based diode exhibited high rectification ratios exceeding 107 and low transition voltages below 0.1 V. When used for energy harvesting via manual bending, the integrated system demonstrated a *V*_oc_ of ∼5.5 V, *I*_sc_ of ∼0.9 μA, and a peak volumetric power density of ∼3.2 mW cm^–3^ (areal density of 0.8 μW cm^–2^). As a biomedical sensor, the device successfully monitored human pulse waves, enabling calculation of pulse rates and estimation of blood pressure.

Ultrathin TENGs are gaining attention as a promising technology for self-powered wearable devices, effectively converting mechanical energy from daily human motions into electricity. Their inherently thin and flexible structure makes them suited for wearable systems that conform gently and unobtrusively to the human body. However, the output performance of TENGs is highly sensitive to the thickness of the triboelectric and dielectric layers. Consequently, to ensure high energy conversion efficiency and stable power generation at ultrathin scales, careful material selection and structural optimization are essential.

In related research, Menge et al. demonstrated a biodegradable and biocompatible ultrathin TENG fabricated from chitosan and alginate using an eco-friendly layer-by-layer self-assembly method (Figure 12c).^164^ The resulting 0.87 μm-thick TENG achieved a remarkable voltage of 474 V and a current density of 36.9 mA m^–2^. This superior performance at the optimal thickness correlated with the film having the highest surface potential of 239.4 mV and the lowest work function of 4.2 eV. The enhancement was attributed to the synergistic effects of the ultrathin multilayer architecture fabricated via layer-by-layer assembly, strong electrostatic interactions between chitosan and alginate layers, and the plasticization by glycerol, which improved flexibility and interfacial contact. These factors collectively enabled efficient charge transfer and accumulation. Compared with previously reported chitosan- and alginate-based TENGs, which typically required much thicker films (50–5000 μm) and yielded lower output (13.5–242 V), the performance of the optimized chitosan/alginate-based TENG stands out for achieving high output.

Next, there is a growing need for ultrathin, self-powered pressure sensors that can respond to low airflow and maintain stable performance. While conventional thick electrostatic sensors are hard to integrate, thinner sensors lack sufficient signal quality at low pressures due to low charge density. The development of high-output, ultrathin, and lightweight self-powered pressure sensors is crucial for practical wearable breath monitoring. Thus, Zhong et al. demonstrated a wireless smart face mask designed for real-time breath condition monitoring, integrating an ultrathin, self-powered TENG-based pressure sensor with a compact wireless readout circuit into a standard face mask (Figure 12d).^165^ The TENG sensor consists of two Au/parylene/Teflon air flow films with a total compressed thickness of ∼5.5 μm and a weight of ∼4.5 mg. It generated a peak open circuit voltage of up to 10 V in response to airflow at 120 Pa and showed a high sensitivity of 0.19 V Pa^–1^ in the low-pressure range (<30 Pa) relevant to human respiration. The device with a readout circuit demonstrated stable wireless operation for up to 8 h, successfully monitoring various breath conditions by wirelessly transmitting data to a smartphone.

Thermoelectric energy harvesters (TEs) generate electricity by converting temperature differences in the surrounding environment into electrical energy. Their performance is fundamentally dependent on the temperature gradient between the two ends of the sensors. Therefore, in ultrathin materials configurations, it becomes difficult to maintain and effectively utilize a significant temperature difference, requiring careful design and performance optimization for integration into wearable devices. In order to overcome these limitations, Li et al. reported a method to enhance both flexibility and thermoelectric performance of composite yarns by applying a continuous ultrathin coating of PEDOT:PSS, modified with dimethyl sulfoxide (DMSO) and 1-ethyl-3-methylimidazolium dicyanamide (ED), onto cotton yarn (Figure 12e).^166^ This P/D/ED coating forms a uniform layer with a thickness of ∼8.78 μm on the yarn surface, in contrast to the penetrating morphology of unmodified PEDOT:PSS. This optimized coating significantly improved the mechanical properties, reducing the modulus from 590 MPa (PEDOT:PSS) to 20 MPa (P/D/ED) and increasing its strain tolerance from 5.5% (PEDOT:PSS) to 31.1% (P/D/ED). The P/D/ED also achieved a high power of 24.7 μW m^–1^ K^–1^. The wearable TE fabric woven from these P/D/ED-coated yarns demonstrated an output power density of 136.1 mW m^–2^ at a temperature difference Δ*T* of 40.8 K.

These results highlight the importance of ultrathin energy harvesters in wearable devices, where minimized thickness enables skin conformity, flexibility, and continuous energy harvesting from body heat. However, despite their potential, several critical challenges remain, including limited power output, long-term mechanical and thermal stability, and integration difficulties with flexible systems. Recent trends address these issues by employing novel materials such as graphene,^168^ MoS_2_,^169^ CNTs,^170^ enhancing stretchability through structural design, and adopting scalable fabrication techniques like printing^146^ and roll-to-roll processing.^171^ These materials technologies hold a promise for applications in self-powered devices, skin-mounted sensors, and smart textiles.

Magnetoelasticity, traditionally associated with rigid metal alloys, has recently been realized in soft silicone composites loaded with micromagnets, enabling strong magneto-mechanical coupling (MC) in tissue-like materials for bioelectronics.^172,173^ In these sensory systems, mechanical stress deforms wavy chains of micromagnets and modulates local magnetic flux, producing a reversible decrease in surface flux density under compression. Coupling this mechanically modulated field to flexible magnetic-induction (MI) coils enables noncontact, self-powered electrical readout suitable for wearable systems. Materials typically embed NdFeB micromagnets in porous silicone.^174^

Recently, Zhou et al. reported a bioinspired magnetoelastic artificial skin (BMAS) that integrates a soft NdFeB/Ecoflex MC layer with stacked MI layers, converting normal and shear inputs into magnetic-flux density variations that are detected by the MI layers (Figure 12f).^167^ The stackable architecture and multiplexed tactile modality on a single location enable force self-decoupling and tactile mapping. Magnetic signaling remained stable under hydraulic pressures up to 25 *m*H_2_O and over 21 days in deionized water and 3.5 wt % NaCl, underscoring environmental robustness. Moreover, MI substrate thickness can be reduced to ∼0.05 mm, which is projected to lower per-layer attenuation from ∼4% to ∼1% and to facilitate vertically stackable, ultrathin magnetoelastic skins. In proof-of-concept underwater tasks, deep-learning-assisted classification achieved ∼95% accuracy for tactile modes and objects, illustrating the modality’s potential for resilient, self-powered sensing systems.

Magnetoelastic skins offer a soft and flexible way to make self-powered sensors. They already appear as thin membranes,^175^ soft fibers,^176^ and textiles^177^ that keep strong magneto-mechanical coupling. To achieve truly ultrathin wearables, both the MC composite and MI substrate must become thinner while the magnetic-flux readout stays stable. The main challenges are long-term durability under repeated strain, robustness in sweat and saltwater, and resistance to electromagnetic noise. With progress in stackable designs, low-power wireless readout, and scalable manufacturing, these systems can become large-area, ultrathin, self-powered wearables,^167^ bioelectronics,^178^ and human-machine interfaces.^179^

### 4.3 Electrophysiological Sensing Materials Designs

Next, electrophysiological signals, commonly referred to as ExG signals, represent a diverse family of bioelectric signals generated by various physiological processes within the human body.^166^ These signals encompass ECG from cardiac electrical activity,^80,180,181^ EMG from skeletal muscle contractions, EOG from eye movements,^182,183^ and EEG from brain neural activity.^13^ ExG signals are characterized by their ability to provide noninvasive measurement of electrical activity between paired electrodes, making them invaluable for clinical diagnostics, research applications, and emerging biosensing technologies.^184–186^

While these signals share the common principle of measuring skin/tissue bioelectric potentials, they exhibit distinct characteristics in terms of anatomical origin, electrode placement requirements, signal amplitude ranges, and frequency bandwidths, with some frequency ranges showing overlapping characteristics between different modalities (Figure 13a).^187,188^ For example, ECG signals operate within a frequency range of 0.5 to 150 Hz, with modern ECG machines recording in this standard bandpass. It is worth noting that high-frequency components up to 250 Hz may be important for detecting specific cardiac conditions.

**Figure 13:**
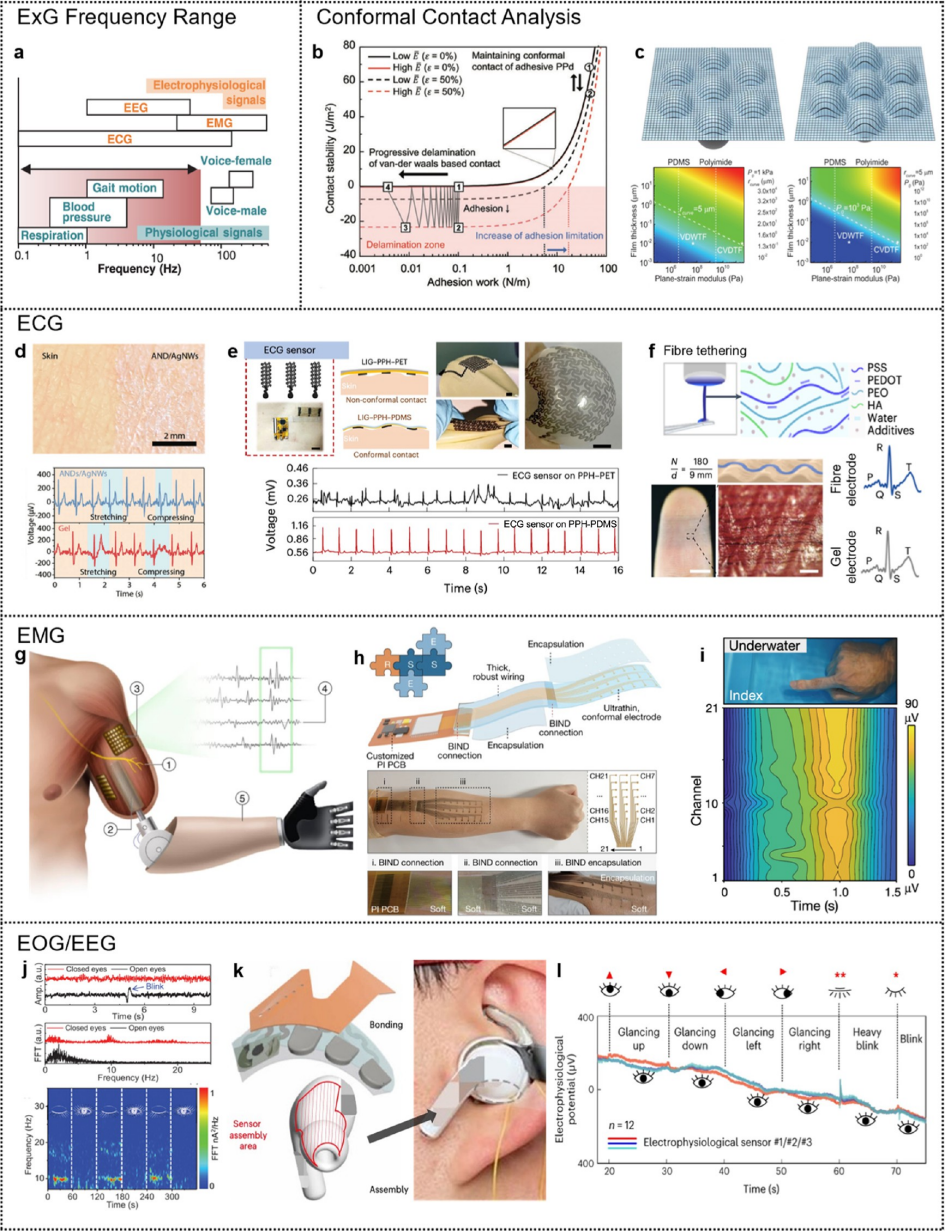
Materials and electrodes for artifact-free electrophysiological signal sensing. (a) Representative frequency ranges of human mechanical and electrophysiological signals. Reproduced with permission from ref 188. Copyright 2022. The American Association for the Advancement of Science. (b) A conformal contact analysis graph depicts the adhesion effect between the skin and electrode interface. Solid lines represent the strain-free state (0%), and dashed lines represent the strain-applied state (50%). The black lines represent a relatively low elastic modulus (*E* = 160 MPa), and the red lines represent a relatively high elastic modulus (*E* = 500 MPa). It demonstrates the mechanisms of general delamination when strain is applied to van der Waals-based contact without any adhesive (squares 1–4) and the maintenance of the conformal contact in PPd by high adhesion (circles 1–2). Reproduced with permission from ref 189. Copyright 2024. Wiley-VCH. (c) (Top) Schematic of conformal contact on a curved surface topography. (Bottom) Contour maps showing the relationship between plane-strain modulus, film thickness, and contact radius at a contact pressure of 1 kPa or maximum contact pressure for a contact radius of 5 mm. Reproduced with permission from ref 15. Copyright 2022. The American Association for the Advancement of Science. (d) (Top) An image of ANDs/AgNWs electrode attached to the skin. (Bottom) ECG signals recorded by ANDs/AgNWs electrodes and gel electrodes during a stretching/compressing cycle. Reproduced with permission from ref 190. Copyright 2024. Wiley-VCH. (e) Conformality of LIG–PPH–PET structure and its attachment to the curvature surface, and robust ECG collection demonstration. Reproduced with permission from ref 86. Copyright 2023 Springer Nature. (f) (Top) A schematic of the fiber tethering process (Bottom) An illustration of a fiber covering of the fingerprints. Comparison of a single ECG signal between the fiber electrode and the gel electrode. Reproduced from ref 191. Available under a CC-BY 40 license. Copyright 2024 Wang et al. (g) Examples of the mechanical and neural interfacing of bionic limbs with the body are targeted muscle reinnervation (1), osseointegration (2), implanted sensors (3), and advanced neural-decoding algorithms (4) that can be combined with modern multiarticulating prosthetic limbs (5) (h) (Top) Schematic of the EMG electrode array, in which the ultrathin electrode module, thick wiring module, PI PCB module, and encapsulation modules are assembled through BIND connections. (Bottom) Photograph of an EMG electrode array on a human arm. Reproduced with permission from ref 192. Copyright 2021 Springer Nature. (i) Photograph intensity contour mapping of 21-channel EMG signals for index finger stretching underwater. Reproduced with permission from ref 81. Copyright 2023 Springer Nature. (j) (Top) Recorded EEG signals and (Bottom) FFT-processed frequency map of the EEG signals. k) (Left) Layer-by-layer structure of in-ear integrated sensors, (Right) the sensors in the ear. Reproduced with permission from ref 15. Copyright 2024 Springer Nature. (l) Transient response to various eye movements recorded with the in-ear integrated sensors. Reproduced from ref 193. Available under a CC-BY 40 license. Copyright 2023 Xu et al.

Among them, EMG signals contain their primary power spectrum within 20 to 500 Hz, with most of the signal power concentrated between 30 and 200 Hz during muscle contraction. The peak frequency typically occurs between 60 and 80 Hz, with the most prominent activity often centered around 70 Hz. EEG frequencies are conventionally analyzed within 0.5 to 70 Hz and are classified into distinct bands. Delta waves occur at 0.5–4 Hz, theta waves at 4–7 Hz, alpha waves at 8–12 Hz, and beta waves at 13–30 Hz. Additional frequency bands include infra-slow oscillations below 0.5 Hz and high-frequency oscillations above 30 Hz, which are gaining clinical importance with advanced digital signal processing techniques. The low-range fundamental frequency of gait typically ranges from 0.8 to 1.0 Hz, corresponding to the stride frequency. Harmonic frequencies for gait mainly appear in the low frequency range below 6 Hz, with the overall gait frequency spectrum generally extending from approximately 0.1 to 10 Hz, reflecting the relatively slow and rhythmic nature of human walking patterns.

The electrode placement strategies reflect the specific anatomical requirements of each modality, ranging from torso, arms, and legs for ECG to uniform scalp placement for EEG using the 10–20 system. Artifact susceptibility varies among modalities, with motion artifacts being particularly problematic for ECG and EMG, while EEG faces challenges from multiple sources, including EMG, EOG, and ECG contamination. The clinical applications span from cardiac diagnostics and heart rate monitoring (ECG) to neuromuscular disease assessment (EMG), sleep studies (EOG), and neurological disorder diagnosis (EEG).

To summarize, Table 2 presents a comprehensive comparison of four primary electrophysiological sensing modalities. As can be seen, the amplitude characteristics vary significantly across modalities, with EMG exhibiting the highest signal amplitudes (1–10 mV), followed by ECG (1–5 mV), while EOG (0.01–0.1 mV) and EEG (0.001–0.01 mV) demonstrate considerably lower amplitudes. The bandwidth requirements also differ substantially, with EMG requiring the broadest frequency range (20–500 Hz) to capture rapid muscle contractions, while EOG operates at the lowest frequencies (0–10 Hz) suitable for slow eye movements.

**Table 2:** General Characteristics of Electrophysiological Sensing Modalities

parameter	ECG	EMG	EOG	EEG
purpose	measures the electrical activity of the heart	measures of electrical activity produced by skeletal muscles	measures eye movement and eye position	records of electrical activity of the brain
electrode placement	torso, arms, and legs	on specific muscles or muscle groups	above, below, and at the side of each eye	uniformly on the scalp, often using the 10–20 system
amplitude	1–5 mV	1–10 mV	0.01–0.1 mV	0.001–0.01 mV
bandwidth	0.05–150 Hz	20–500 Hz	0–10 Hz	0.5–70 Hz
common applications	diagnosis of heart conditions like ischemia, arrhythmia, and conduction defects. Monitoring heart rate variability	diagnosis of neuromuscular diseases. Muscle function analysis. Prosthesis arm, leg	sleep studies (PSG) for eye movement analysis. Eye movement artifact correction in EEG	diagnosis of epilepsy, sleep disorders, and brain tumors. Cognitive and psychological research
signal characteristics	large amplitude, low-frequency signals. R-peaks for heart rate detection	high-frequency signals due to Muscle contractions	low amplitude, very low-frequency signals. Sensitive to eye movements	very low amplitude, a wide range of frequencies. Sensitive to brain activity and artifacts
artifacts	motion artifacts, powerline interference	movement artifacts, electrical noise	eye blinks, eye movements	EMG, EOG, ECG artifacts

The diverse signal characteristics and electrode placement requirements highlighted in this comparison underscore a critical challenge common to all electrophysiological sensing modalities: achieving and maintaining optimal electrode-skin interface quality. The significant variation in signal amplitudes, particularly the extremely low amplitudes of EEG signals (0.001–0.01 mV), combined with the susceptibility to motion artifacts across all modalities, compromises practical developments and directly requests the fundamental importance of conformal contact between designed materials, electrodes, and skin.

Overall, acquiring these signals robustly presents significant challenges. In most settings, electrical or optical signals must pass through the skin, which has high electrical resistivity, creating a natural barrier to precise monitoring.^194^ This issue is compounded in long-term monitoring scenarios where subjects perform daily activities, as free movement introduces significant noise and motion artifacts into the raw data. As demonstrated in recent research, conformal contact with skin can reduce interface impedance and enhance signal quality, while inadequate contact leads to high interface impedance. This interface quality becomes particularly critical when considering the low-amplitude nature of bioelectric signals and their vulnerability to noise contamination, establishing conformal contact as a prerequisite for robust electrophysiological signal collection across all sensing modalities and at challenging dynamic biological surfaces.

For instance, motion artifacts and baseline wandering typically reside in the low-frequency range of 0–3 Hz, which can overlap with and corrupt the desired physiological signals.^188^ While various innovative digital and AI-assisted filtering techniques have been developed to extract valuable information postcollection,^195^ the most effective strategy is to minimize noise at the initial signal acquisition stage. To achieve this, establishing and maintaining conformal contact between the sensing electrode and the skin is of primary importance. Existing wearable bioelectronic sensors can be susceptible to motion artifacts because they often lack the necessary adhesion and conformal interfacing to handle skin deformation.^185^

This critical issue can be resolved by designing ultrathin, flexible, and stretchable “skin-like” or “epidermal” electronics from novel materials with matching mechanical properties—such as low Young’s modulus, dynamic properties, and tailored bending stiffness. Such material designs allow the sensory films to intimately cover, adhere, and laminate to the skin’s surface, ensuring stable contact even on dynamically deformed and wrinkled areas. This close skin adherence minimizes voids at the interface, increases the effective contact area, and secures a continuous, high-fidelity electrical connection, paving the way for advanced wearable systems like ultrathin dry electrodes and “drawn-on-skin” electronics capable of accurate, long-term monitoring with high immunity to motion artifacts.^67,196^

A lot of research efforts have been made to understand under what conditions the conformal contact can be achieved. For example, Shin et al. analyze conformal contact by calculating the total energy ( ${\mathrm{U̅}}_{total}$ ) of the electrode-skin system, which includes bending energy, skin elastic energy, adhesion energy, and membrane energy.^189^ Figure 13b illustrates how contact stability (negative of total energy) depends on adhesion work (γ) and elastic modulus ( ${\mathrm{E̅}}_{c}$ ) under applied strain.^189^ The graph shows that conformal contact is maintained when stability >0, while delamination occurs when stability <0. Higher adhesion and lower elastic modulus improve stability, enabling the electrode (with ${\mathrm{E̅}}_{c}$ = 160 MPa and γ = 51.4 N/m) to resist delamination even under 50% strain, unlike conventional electrodes like Au or pristine PEDOT:PSS films.

For instance, Yan et al. analyzed conformal contact through mechanical energy minimization and FEA modeling, focusing on balancing adhesion, elastic modulus, and thickness to achieve stable interfaces.^15^ Figure 13c illustrates the conformal adaptation process of a stretchable membrane on spherical topographies, showing how grid deformation accommodates strain under contact pressure. Also, the color maps quantify this relationship via contour maps: the left one reveals that reducing film thickness and plane-strain modulus (e.g., VDWTFs’ 47.3 MPa vs PI’s 2.8 GPa) increases contact radius at a fixed pressure, while the right one demonstrates that lower modulus and thickness minimize required contact pressure (e.g., ≤1 kPa for skin compatibility). These analyses establish that ultrathin VDWTFs avoid mechanical mismatch with biological tissues by enabling strain dissipation through nanosheet sliding rather than lattice deformation.

Finally, Kim et al. analyzed conformal contact by calculating the strain energy and adhesion work required for ultrathin GaN membranes to laminate onto skin topography.^67^ The authors model the balance between bending energy and adhesive energy from the PDMS patch, determining that thinner GaN films (≤300 nm) minimize strain energy while maintaining adhesion. 200 nm-thick GaN achieves conformal contact by reducing mechanical mismatch with skin (modulus ∼130 kPa), whereas thicker films (e.g., 1800 nm) cannot deform to match microscale skin features. This theoretical framework is validated experimentally, where 200 nm GaN adheres seamlessly to skin.

#### 4.3.1 ECG Monitoring Applications

An ECG captures the small electrical changes on the skin that are a consequence of cardiac muscle depolarization and repolarization during each heartbeat.^197^ The resulting waveform shows the rate and rhythm of the heartbeats, as well as the strength and timing of the electrical signals as they travel through the different chambers of the heart.^198^ This data provides a detailed look at the orderly progression of electrical activity that coordinates the heart’s pumping action. In a clinical setting, an ECG is a fundamental and often initial diagnostic tool for evaluating a patient’s heart health, such as irregular heartbeats (arrhythmias), coronary artery disease, and a heart attack.

Novel approaches have been reported for making thin-film electrodes for stable ECG monitoring. To create the thin film structure, Zhao et al. use a spin-coating process to deposit a solution of aramid nanofibers derived from Kevlar onto a wafer (Figure 13d).^190^ A subsequent reprotonation step solidifies the film, resulting in a transparent, robust, and ultrathin aramid nanodielectric (AND) layer. For conformal contact, the film’s minimal thickness reduces its bending stiffness, allowing it to naturally adhere to the skin topology. To improve this adhesion for long-term wear, a biocompatible PU adhesive is applied, which increases the bonding force to the skin without harming its breathability. For ECG signal collection, the film is made conductive by spin-coating a network of AgNWs onto its surface. Also, its conformal contact minimizes motion artifacts and maintains performance even during skin deformation or in the presence of sweat.

In another study, Lu et al. reported an ultrathin conductive nanocomposite using a cryogenic transfer approach at −196 °C, which enabled the transfer of LIG patterns onto an adhesive poly­(vinyl alcohol)-phytic acid-honey (PPH) hydrogel with a thickness of only 1.0–1.5 μm (Figure 13e).^86^ This ultrathin hydrogel serves as both an energy dissipation interface and an out-of-plane electrical pathway, allowing deflected crack formation in the LIG that enhances stretchability over 5-fold compared to traditional LIG-PDMS composites. Also, it is demonstrated that high-quality ECG signals can be obtained from the PPH–PDMS films while transferring LIG onto PPH–PET films, resulting in unsteady and fluctuating signals due to the nonstretchable PET layers. The ultrathin PPH film showed negligible interference with signal quality, as evidenced by the on-skin impedance between LIG electrodes being similar to that of disconnected electrodes.

Furthermore, Wang et al. fabricated thin, substrate-free bioelectronic fibers from a solution containing PEDOT:PSS, hyaluronic acid, and poly­(ethylene oxide) (Figure 13f).^191^ They employed an “orbital spinning” technique that draws strands from this solution to tether them directly onto biological surfaces, such as a fingertip, in a contactless and mask-free process. To achieve conformal contact with the skin, the fibers were spun in a wet state, allowing residual water to create dominant Wenzel-like contact with the surface. This ensures the fibers form intimate attachments that conform to microtopographies like fingerprint ridges (Figure 13f). These fiber networks are used to create on-skin electrodes. An array of bioelectronic fibers is deposited on the index fingers of both hands, which serve as working and counter electrodes. This configuration successfully acquires high-quality biopotential signals, producing ECG readings consistent with those from standard gel electrodes.

Beyond these solid- and fiber-based strategies, emerging liquid-based bioelectronic systems offer a new frontier for achieving fully conformal ECG monitoring. In particular, permanent fluidic magnets (PFMs) form three-dimensional oriented and ramified magnetic networks that flow into skin wrinkles and microgrooves, minimizing impedance mismatch and motion artifacts while preserving long-term magnetic stability.^199^ When coupled with soft receiver coils, PFM-based systems transduce cardiac-induced microdeformations into magnetic flux changes, enabling continuous and motion-robust ECG and pulse wave monitoring.^200^ Similarly, liquid acoustic sensors have demonstrated enhanced skin conformity and self-filtering properties for high-fidelity signal acquisition even in noisy or dynamic environments.^201^ Together, these advances suggest that liquid-state materials can complement or surpass solid thin-film approaches for next-generation bioconformal ECG sensors.

#### 4.3.2 EMG Monitoring Applications

EMG is the electrical activity generated by skeletal muscles.^202^ Motor neurons transmit electrical signals that cause muscles to contract, and an EMG instrument uses electrodes to capture these signals. EMG has a wide range of applications beyond its common use as a diagnostic tool for neuromuscular disorders. In production engineering, it is used for ergonomic assessments of workstations and tools, as well as monitoring fatigue and physical strain or enabling gesture recognition to control robotic systems.

Especially for patients with amputations, researchers are pursuing invasive EMG sensors primarily to overcome the significant limitations of surface EMG that compromise signal quality and reliability for delicate control of a prosthesis (Figure 13g). For this application, invasive/implantable EMG sensors offer several key advantages: they can measure EMG cross-talk-free signals, provide reliable signals at diverse locations, and enable access to both superficial and deep muscles.^192^ A biphasic, nanodispersed (BIND) interface has been fabricated from an interpenetrating nanostructure of a self-adhesive polymer and metal nanoparticles, which provides continuous mechanical and electrical pathways, resulting in multipathway connections.^81^ This interface enables the simple “plug-and-play” assembly of soft, rigid, and encapsulation modules to create complex, highly stretchable hybrid electronic devices (Figure 13h). This system demonstrated effective performance by simply switching the “soft module” part in the system. The key to eliminating noise was the robustness of the BIND connections linking the ultrathin electrode, the wiring, and the rigid circuit board. When subjected to mechanical interference like direct pressure from a tweezer clamp or 50% stretching, these BIND connections maintained stable electrical contact that allowed the BIND electrode to maintain a consistently high signal-to-noise ratio, effectively preserving the clarity of the EMG signal even in underwater conditions (Figure 13i).

#### 4.3.3 EOG/EEG Monitoring Applications

EOG is the resting potential between the front and back of the human eye, which can be exploited for eye monitoring.^203^ By placing electrodes around the eyes, the EOG technique records this corneo-retinal potential to track eye movements.^204^ The human eye acts as a dipole, with a positive charge at the cornea and a negative charge at the retina, creating a potential difference that changes as the eye moves. EEG is the signal that is recorded from the brain’s electrical activity.^205^ Electrodes are placed on the scalp to detect the tiny electrical charges produced by brain cell activity.^206^

It is worth noting that EOG and EEG are electrophysiological monitoring that are often used together, particularly in assessing alertness and in sleep studies.^207^ A significant aspect of their relationship is signal interference or crosstalk. Eye movements recorded by EOG can appear as artifacts in EEG recordings, which can affect data interpretation. Conversely, EEG signals can be coupled into EOG recordings because the electrodes are placed in proximity. Modern hybrid systems are starting to utilize EOG signals as an additional control input alongside EEG.^206^

In related research, Yan et al. reported the EEF sensors with the use of staggered 2D nanosheets made of MoS_2_.^15^ These films with the individual nanosheets to sliding against possess the exceptional stretchability, malleability, and adaptability, enabling them to form a seamless, conformal interface. With this material, they designed a transistor and attached it to the head to measure the EEG signal (Figure 13j). Then, Xu et al. developed a fully integrated, user-generic sensor array designed to be worn unobtrusively in the ear for the simultaneous and continuous monitoring of both brain activity and metabolic biomarkers in sweat.^193^ To measure EEG and EOG signals, the sensor employs a set of stretchable Ag electrodes: three electrophysiological sensors placed within the ear canal, a reference (REF) electrode on the concha cymba, and a driven right leg (DRL) electrode on the concha cavum to suppress common-mode interference (Figure 13k). The 3D electrophysiological electrodes ensure robust, conformal contact within the ear canal, accommodating anatomical variations among users. The device’s efficacy in capturing EEG signals was demonstrated by detecting alpha modulation and responses to specific acoustic frequencies. This device also captures EOG signals generated by eye movements like blinking and glancing and reduces interference between EEG and EOG signals, improving reliability for real-world brain-state monitoring (Figure 13l).

### 4.4 Optical Sensors Applications

Optical sensors offer immediate and intuitive visual feedback in response to various physical stimuli, enabling natural and real-time monitoring. This real-time visualization of stimuli enhances immersion and usability, making optical systems highly effective for applications in wearable electronics (Figure 14).^208,209^

**Figure 14:**
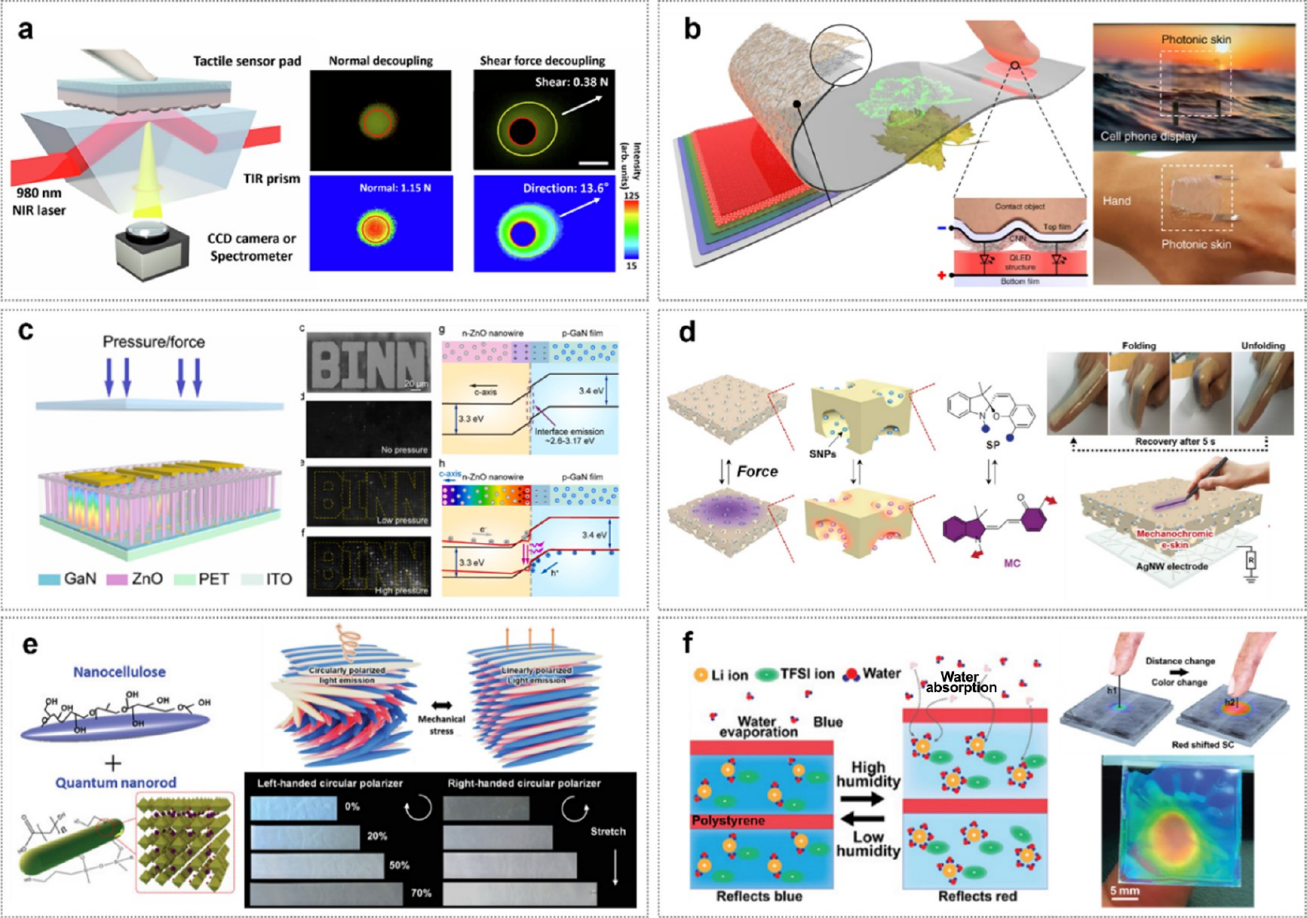
Ultrathin Optical sensors and devices. (a) UCN-based optical tactile sensor structure (left), and machine learning-based force decoupling system (right). Reproduced from ref 215. Available under a CC-BY 40 license. Copyright 2024 Son et al. (b) Schematic of the photonic skin structure and photograph of the laminated on various 3D surfaces. Reproduced from ref 216. Available under a CC-BY 40 license. Copyright 2020 Lee et al. (c) Schematic of a working mechanism and photograph of sensor light emission by piezophototronic effect. Reproduced with permission from ref 217. Copyright 2019 Elsevier. (d) Schematic of structure and working mechanism of mechanochromic sensors (left), and photographs of application in E-skin and spatiotemporal detection (right). Reproduced with permission from ref 218. Copyright 2019 Wiley-VCH. (e) Schematic of a bioinorganic chiral nematic organization (left), and mechanisms and photographs of the change in photonic appearance in response to an increase in mechanical strain (right). Reproduced with permission from ref 219. Copyright 2021 Wiley-VCH. (f) Mechanism of crystallization change in PC film by humidity change (left), and photographs of a touchless real-time interactive display. Reproduced from ref 220. Available under a CC-BY 40 license. Copyright 2020 Kang et al.

Unlike conventional tactile displays that require pixelated sensor arrays, which often lead to increased circuit complexity and crosstalk between sensing elements,^210^ novel optical systems leverage intrinsic material responses such as opto-tactile coupling,^211^ piezophototronics,^212^ mechanochromism,^213^ and photonic crystal modulation^214^ to enable continuous, direct, and power-efficient visualization. Opto-tactile sensors transduce mechanical stimuli (pressure, shear, dynamic touch) into optical signals, enabling real-time, quantitative mapping of tactile events. Using an ultrathin form factor further improves their performance. Ultrathin, skin-inspired designs allow for high sensitivity and the decoupling of force components from a single optical image.

A representative example is the upconversion nanocrystal (UCN)-based optical tactile sensor (Figure 14a).^215^ This sensor utilizes a multilayer structure composed of a stress concentration layer that mimics the undulating interface of human skin, and a tactile layer embedded with UCNs, which are excited via frustrated total internal reflection at the interface with a prism. When force is applied, the stress concentration layer amplifies and localizes both normal and shear forces, which are then transduced into spatially resolved luminescence.

Notably, this sensor enables instantaneous and quantitative decoupling of vertical (normal) and lateral (shear) forces from a single image in real time. The ultrathin design, with microhemispheres patterned in the PDMS layer and a total thickness in the order of hundreds of micrometers, ensures excellent conformability to curved or soft surfaces, minimizes signal distortion or loss, and offers high spatial–temporal resolution even under rapid or complex tactile interactions. In another approach, Lee et al. developed an ultraflexible and transparent opto-tactile sensor by combining two ultrathin films: a transparent pressure-sensing film based on a cellulose/nanowire network with ultrahigh sensitivity (>5000 kPa^–1^) and a quantum dot-based electroluminescent film (Figure 14b).^216^ These films conform perfectly to complex 3D surfaces, transducing spatial pressure into a continuous conductivity distribution and enabling analog, pixel-less pressure imaging with a resolution exceeding 1000 dpi. The total device thickness is about 3 μm, which minimizes distortion in pressure mapping, ensures high cyclic stability, and supports fast response time (<1 ms).

Next, piezophototronic devices exploit the coupling of piezoelectricity, semiconductors, and photonics to enable direct visualization of mechanical stimuli by inducing piezoelectric polarization with enhanced electroluminescence. In this field, Peng et al. demonstrated a flexible p-GaN/n-ZnO nanowire LED array that operates via the piezo-phototronic effect, achieving ultrahigh-resolution pressure mapping by directly modulating electroluminescence intensity (Figure 14c).^217^ Unlike conventional displays based on planar thin film or bulk materials, this device forms pixels using vertically aligned ZnO nanowires grown on a thin (5 μm) flexible GaN film with each ZnO nanowire acting as an independent light-emitting pixel, enabling parallel optical readout. The nanoscale geometry allows for a spatial resolution of 2.6 μm, far surpassing that of human skin and traditional tactile sensors, and ensures fast response (180 ms) even after thousands of pressure cycles. The ultrathin, flexible design further enhances mechanical compliance, transparency, and robustness, allowing to maintain stable performance under deformation.

Next, mechanochromic polymer sensors are based upon materials that undergo visible color or fluorescence changes in response to mechanical stimuli. These systems enable direct, power-free visual mapping of stress or deformation. Park et al. developed a nanoparticle-in-micropore structure embedded within a porous PDMS matrix, combining spiropyran mechanophores with silica nanoparticles (Figure 14d).^218^ This design facilitates a soft, stretchable, and thin composite film capable of concentrating stress, thereby dramatically enhancing sensitivity and allowing for reversible color changes with rapid recovery and high durability for repeated deformations. The compliant nature of the material ensures that even small, localized forces are efficiently transmitted to the mechanophores, enabling power-free visualization of force distribution and dynamic events such as writing or vibration.

Finally, photonic crystal-based sensors employ periodic nanostructures whose optical properties change in response to mechanical deformation. Changes in spacing or refractive index result in visible color shifts, enabling these systems to function as opto-mechanical sensors. Thus, Kang et al. developed a chiral photonic elastomer by coassembling quantum nanorods and cellulose nanocrystals into a chiral nematic photonic structure within a physically cross-linked PU elastomeric matrix (Figure 14e).^219^ This architecture allows for dynamic and reversible modulation of both structural color and polarization state in response to mechanical deformation. The ultrathin and flexible design not only enables conformal lamination onto diverse substrates but also provides mechanical resilience and anisotropic elastic properties, including a near-zero in-plane Poisson’s ratio.

In a separate study, Kang et al. also reported a 3D touchless structural color sensing display based on an ultrathin solid-state block copolymer photonic crystal film (Figure 14f).^220^ This ultrathin, flexible film incorporates a chemically cross-linked hydrogel network, yielding a modulus of a few hundred MPa, providing sufficient elasticity. The nanoscale periodicity of the PC film ensures sharp and well-defined photonic stop bands, resulting in vivid and pure structural colors with minimal scattering, even after repeated mechanical bending or transfer. When the ultrathin film is exposed to humidity, such as from a nearby finger presence, the film undergoes rapid swelling along its nanostructured layers, modulating the local refractive index and periodicity and thus reversibly changing color for visualization of finger proximity.

## 5 Conclusion and Outlook

In this review, we presented a comprehensive overview of the key materials developments for applications of ultrathin soft wearable sensors in the domains of electrophysiological monitoring and tactile sensing. By achieving intimate contact with the skin and matching its mechanical, topographical, and physiological characteristics, these sensors enable accurate, continuous, and noninvasive signal acquisition, offering transformative potential across healthcare, sports, and human-machine interaction. We discussed the key material and structural design strategies that support the sensing performance and reliability of ultrathin wearable systems. These approaches include the use of intrinsically soft and conductive materials, hybrid nanocomposites, and porous or nanofiber architectures to ensure conformability, breathability, and functional robustness. Special attention was given to interface engineering techniques such as smart adhesives, drawn-on-skin electronics, and e-tattoos, which allow reliable integration of devices onto complex and dynamic skin surfaces. Finally, we summarize the use of material sensors for various applications, including tactile sensors, energy harvesters, electrophysiological sensors, and optical sensors.

Overall, truly ultrathin wearable sensors represent a convergence of advanced materials science and biomedical engineering, yet several technical and translational challenges remain to enhance mechanical durability, biocompatibility, environmental stability, and scalability in order to fully realize their practical potential. We suggest that future research efforts should be undertaken by materials and sensory communities in order to facilitate further progress in the field, as briefly summarized below. The emergence of novel ultrathin and ultraflexible soft functional materials for skin-integrated wearable sensors marks a pivotal shift from conventional rigid component (metals, semiconductors) monitoring systems toward truly adaptive, skin-matching and conformal materials technologies that can be seamlessly integrated with the various parts of the human body. Among critical tasks under consideration, we can mention.

### 5.1 Seamless Materials-Driven Mechanical Integration across all Biological and Synthetic Interfaces

Significant progress has been made in designing sensors that conform to the mechanical properties of human skin. However, mechanical mismatches still exist at the interfaces between the sensing materials and the electrode, and between the metal electrodes and the external data acquisition system. These discontinuities often result in motion artifacts, signal degradation, or device delamination during dynamic movements. Future work should further address mechanical compliance not only at the skin-sensor interface but also throughout the entire signal acquisition chain to ensure robust, stable sensor performance in real-world environments.

### 5.2 Pathways toward Fully Human-Integrated, Standalone, Seamless Sensors and Complete Sensor Systems

While ultrathin sensors can reliably detect various biosignals, most systems still rely on bulky external instrumentation units to process and interpret the sensing data. This “bulky” device design often necessitates long, rigid wiring between the sensor and external modules, which compromises the universal wearability, long-term performance, and user mobility. Developing a fully integrated system, combining sensing materials, signal processing, signal transmission, wireless transmission, and power management within a single compact materials/structure platform, remains an essential but unmet challenge. Achieving such full-system integration will require co-optimization of sensing materials, electronics, and form factors while maintaining skin conformity, self-powering, or low power consumption.

### 5.3 Development of Multimodal, Decoupled and Universal Sensing Platforms

For practical use in real-time health monitoring and diagnostics, a single device must be capable of detecting multiple physiological signals—such as electrophysiological, thermal, optical, and biochemical cues—simultaneously. However, integrating multiple sensing modalities into one ultrathin platform presents challenges in maintaining cross-signal fidelity and avoiding interference. Signal decoupling strategies, such as spatial, material-based, or frequency-domain separation, must be carefully engineered to ensure accurate and independent measurements for each functional modality.

### 5.4 Further Efforts toward Scalability, Reliability, and Commercial Translation

Although proof-of-concept demonstrations have shown impressive performance, large-scale manufacturing, long-term reliability, and operation, especially under extreme environmental conditions (e.g., high humidity, sweating, underwater, or during intense activity) are still not fully addressed. Ensuring consistent sensor performance over extended periods, especially for applications involving continuous monitoring under real-life habits and conditions, is critical for practical daily, indoor and outdoor conditions, and clinical adoption. Additionally, scalable and readily available synthesis, fabrication, and transfer methods, novel sensor materials, device standardization, and regulatory approval pathways must be further addressed and developed to transition novel ultrathin wearable sensors from laboratory settings to commercially viable products.

In conclusion, the challenges outlined in this review form a strategic roadmap for advancing the next generation of ultrathin wearable devices, as graphically summarized in Figure 15. Addressing these hurdles will not only deepen our scientific understanding of human physiology but also catalyze transformative innovations in personalized healthcare, real-time diagnostics, and human-machine symbiosis. Furthermore, we foresee that universal ultrathin human-integrated sensors are poised to extend far beyond terrestrial applications.

**Figure 15:**
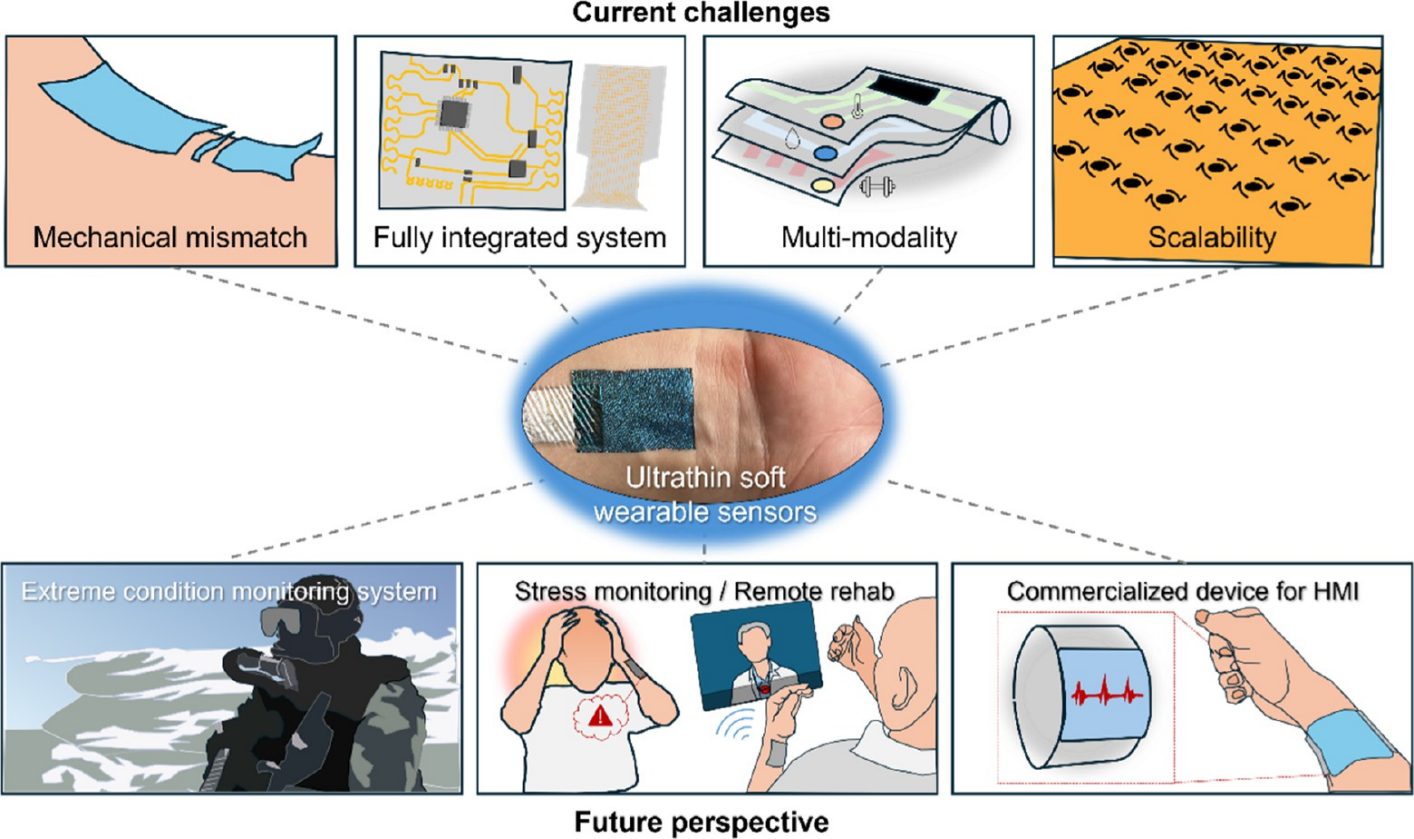
Current challenges and future perspectives of ultrathin wearable sensors. Current challenges include mechanical mismatch with human skin or sensor–electrode interfaces, integration of sensors with circuit boards/hard electronics, fabrication of multimodal sensors, and fabrication of scalable, commercially available sensors. For a future perspective, ultrathin soft wearable sensors have the potential to be applied in extreme conditions, remote healthcare systems, and commercialized personal healthcare devices.

For example, in extreme marine environments, skin-conformal bioelectronics can enable diver health monitoring under pressure and salt exposure. In aerospace and space missions, breathable, radiation-tolerant sensors can monitor astronauts’ vitals without encumbering bulky suits. Furthermore, far-reaching applications in very long journeys in space and on planets with extremely challenging human-unfriendly conditions will demand even more advanced materials for responsive and protective skin, eye, muscles, or brain-integrated sensors well beyond the abilities of current materials technologies.

Back on Earth, seamless sensor integration allows for real-time stress monitoring in athletes, remote rehabilitation in the elderly, and early detection of infection or dehydration in combat zones. These futuristic visions will demand further innovations in self-powered operation, multimodal sensing, autonomous data processing, and AI-integrated closed-loop feedback and human monitoring. To this end, innovation-driven companies like MC10, Quad Industries, iRhythm, Epicore Biosystems, and Profusa can successfully translate academic research into market-ready products targeting healthcare, sports, extreme explorations, and consumer wellness. Furthermore, tech giant companies such as Apple, Meta, and Samsung are actively investing in skin-integrated sensors to enhance wearable product lines like smartwatches and AR/VR headsets, signaling strong industrial interest and commercial traction.

Realizing this vision in near future will require sustained interdisciplinary research activities and deep collaboration across materials science, flexible electronics, biomedical engineering, extreme outdoor activities, space presence, and clinical research to transition these groundbreaking materials technologies from the laboratory to commercial developments for everyday life utilization.
